# CDK4/6 initiates Rb inactivation and CDK2 activity coordinates cell-cycle commitment and G1/S transition

**DOI:** 10.1038/s41598-022-20769-5

**Published:** 2022-10-07

**Authors:** Sungsoo Kim, Alessandra Leong, Minah Kim, Hee Won Yang

**Affiliations:** 1 Department of Pathology and Cell Biology, Columbia University, New York, NY 10032 USA; 2 Herbert Irving Comprehensive Cancer Center, Columbia University, New York, NY 10032 USA

**Keywords:** Checkpoint signalling, Cell-cycle exit, Checkpoints

## Abstract

External signaling controls cell-cycle entry until cells irreversibly commit to the cell cycle to ensure faithful DNA replication. This process is tightly regulated by cyclin-dependent kinases (CDKs) and the retinoblastoma protein (Rb). Here, using live-cell sensors for CDK4/6 and CDK2 activities, we propose that CDK4/6 initiates Rb inactivation and CDK2 activation, which coordinates the timing of cell-cycle commitment and sequential G1/S transition. Our data show that CDK4/6 activation induces Rb inactivation and thereby E2F activation, driving a gradual increase in CDK2 activity. We found that rapid CDK4/6 inhibition can reverse cell-cycle entry until CDK2 activity reaches to high levels. This suggests that high CDK2 activity is required to initiate CDK2-Rb positive feedback and CDK4/6-indpendent cell-cycle progression. Since CDK2 activation also facilitates initiation of DNA replication, the timing of CDK2-Rb positive feedback is coupled with the G1/S transition. Our experiments, which acutely increased CDK2 activity by cyclin E1 overexpression, indicate that cells commit to the cell cycle before triggering DNA replication. Together, our data suggest that CDK4/6 inactivates Rb to begin E2F and CDK2 activation, and high CDK2 activity is necessary and sufficient to generate a bistable switch for Rb phosphorylation before DNA replication. These findings highlight how cells initiate the cell cycle and subsequently commit to the cell cycle before the G1/S transition.

## Introduction

Entry into the cell cycle is tightly regulated in multicellular organisms, and dysregulation of this process can lead to cancer^1^ and degenerative diseases^2^. Cell-cycle entry is regulated by the retinoblastoma protein (Rb) and cyclin-dependent kinase 4 and 6 (CDK4/6)^3–6^. During quiescence, Rb serves as a transcriptional co-repressor that binds E2F proteins and represses their transcriptional activities^5,7^. In the canonical model of cell-cycle entry, mitogenic signaling upregulates expression of cyclin D which directly binds to and activates CDK4/6. In turn, active CDK4/6 induces partial phosphorylation of Rb to disrupt the Rb/E2F interaction and to initiate E2F activity and expression of CDK2 activators, cyclin E/A^5,7–12^. CDK2 activation triggers full Rb phosphorylation (hyperphosphorylation) and generates a CDK2-Rb positive feedback loop, creating a bistable switch for Rb phosphorylation^5,7–9,13^. This CDK2-mediated positive feedback triggers cell-cycle commitment (the restriction point), after which cells proceed through the cell cycle regardless of mitogen availability^6,14–16^.

Previous experiments demonstrated that cyclin D-CDK4/6 and cyclin E/A-CDK2 induce Rb phosphorylation^9,13,17–20^. However, the timing of Rb/E2F pathway regulation by CDK4/6 and CDK2 in relation to cell-cycle commitment remains unclear. A recent study showed Rb hyperphosphorylation in mouse embryonic fibroblasts (MEFs) lacking cyclin E and A that can be suppressed by treatment with the CDK4/6 inhibitor palbociclib, suggesting that CDK4/6 alone is enough to induce Rb hyperphosphorylation and E2F activation^21^. Conversely, another study performed a systematic analysis of Rb phosphorylation patterns and revealed that CDK4/6 induces only Rb monophosphorylation, while supporting the idea that CDK4/6 facilitates cell-cycle entry^22^. A following study further characterized different monophosphorylation forms of Rb that are associated with different transcriptional outputs^23^. Other studies showed that palbociclib treatment or stress induction, which causes rapid CDK4/6 inhibition^24^, can reverse Rb phosphorylation even after CDK2 activation until inactivation of the anaphase-promoting complex/cyclosome-Cdh1 (APC/C^Cdh1^) at the onset of S phase^21,25,26^. Since cyclin A is degraded by APC/C^Cdh1^^27,28^, this raises a question whether cyclin E-CDK2 can phosphorylate Rb in cells or whether other regulatory mechanisms are required to initiate CDK2-Rb positive feedback for CDK4/6-independent cell-cycle progression^29^. Given recent studies revealing that FDA-approved CDK4/6 inhibitors, palbociclib, ribociclib, and abemaciclib, can indirectly suppress CDK2 activity by redistributing the CDK interacting protein/kinase inhibitory protein (Cip/Kip) family, notably p21, to CDK2^30,31^, the indirect effects of palbociclib on CDK2 activity may also explain the reversibility of Rb phosphorylation in cells with low CDK2 activity. Nevertheless, it remains elusive whether (1) cyclin E-CDK2 can phosphorylate Rb in cells, (2) additional mechanisms in S phase are required for CDK2 to induce Rb phosphorylation, (3) CDK4/6 inhibitors indirectly cause the reversibility of CDK2 activation and cell-cycle entry, and (4) high CDK2 activity is necessary to trigger CDK2-Rb positive feedback.

Here, using CDK4/6 and CDK2 reporters, we examined CDK regulation of the Rb/E2F pathway in relation to cell-cycle commitment and the G1/S transition at the single-cell level. By analyzing kinetics of Rb phosphorylation and E2F activation, our results indicate that active CDK4/6 rapidly drives Rb inactivation and E2F activation regardless of CDK2 activation kinetics. Importantly, using p21/p27/p57 triple knockout (tKO) cells and inducible overexpression of cyclin E1, our data show that high cyclin E1-CDK2 activity is necessary and sufficient to phosphorylate Rb before promoting DNA replication. Thus, CDK2 activity coordinates the timing of cell-cycle commitment and the G1/S transition. Collectively, our data demonstrate that CDK4/6 activity is sufficient to initiate E2F activity and that high CDK2 activity is required to initiate CDK2-Rb positive feedback and CDK4/6-independent cell-cycle progression.

## Results

### CDK4/6 activation induces Rb inactivation

**Figure 1:**
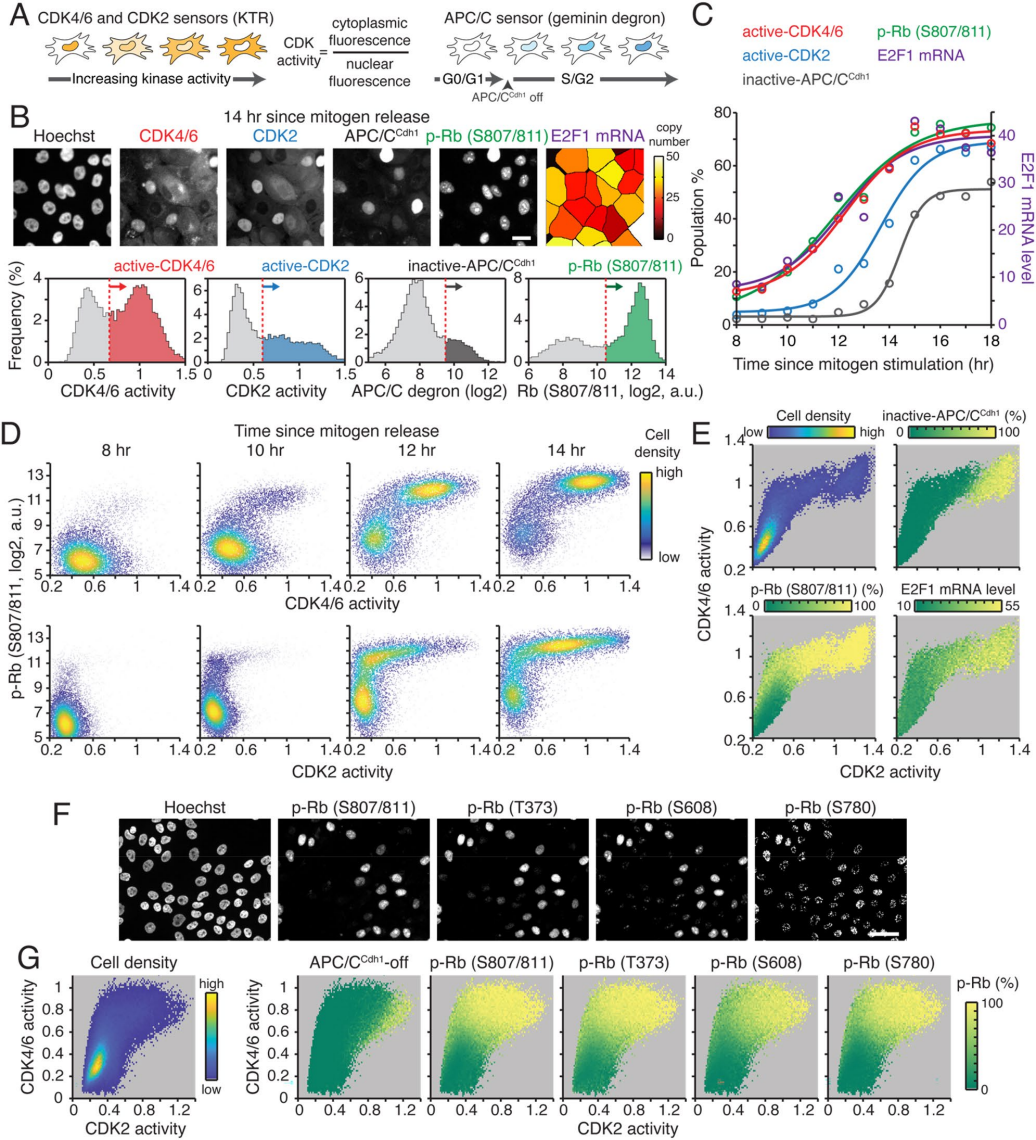
CDK4/6 activation is tightly coupled with Rb phosphorylation and E2F1 mRNA induction. (**A**) Schematics of CDK4/6, CDK2 (left), and APC/C^Cdh1^ (right) reporter. Active CDK4/6 and CDK2 phosphorylate and induce translocation of reporters to the cytoplasm. As APC/C^Cdh1^ is inactivated, intensity of the reporter starts increasing. (**B**) Top, representative images of CDK4/6, CDK2, and APC/C^Cdh1^ reporters, phosphorylation of Rb (p-Rb, S807/811) immunostaining, and mRNA FISH for E2F1 mRNA in the same MCF-10A cells. Scale bar is 20 µm. Bottom, histograms of CDK4/6, CDK2, and APC/C^Cdh1^ activities and p-Rb at S807/811. Red dotted line indicates a threshold used to classify active CDK4/6 and CDK2, inactive APC/C^Cdh1^, and p-Rb populations. After 48 h mitogen removal, cells were stimulated with mitogens for 14 h prior to fixation. (**C**) Scatter plot of the percentage of cells with active CDK4/6 and CDK2, inactive APC/C^Cdh1^, p-Rb at S807/811, and averaged E2F1 mRNA levels in MCF-10A cells as a function of time after mitogen stimulation. Solid line represents sigmoidal best-fit line. (**D**) Single-cell correlation of p-Rb at S807/811 versus CDK 4/6 activity (top) and CDK2 activity (bottom) in MCF-10A cells at various time points after mitogen stimulation (8, 10, 12 and 14 h). Cell density is color-coded. (**E**) Three-dimensional activity map of CDK4/6 versus CDK2 activity in MCF-10A cells where cell density, the percentage of inactivated APC/C^Cdh1^, the percentage of p-Rb at S807/811, and E2F1 mRNA levels are color-coded. (**F**,**G**) Representative images of multiplexed staining for p-Rb (S807/811, T373, S608, S780) in MCF-10A cells (**F**). Scale bar is 20 µm. Three-dimensional activity map of CDK4/6 versus CDK2 activity where cell density, the percentage of inactivated APC/C^Cdh1^, and the percentage of p-Rb at S807/811, T373, S608, or S780 are color-coded (**G**). After 48 h mitogen removal, MCF-10A cells were stimulated with mitogens for 12 h prior to fixation.

To understand the sequence of events in cell-cycle entry and in Rb/E2F pathway regulation, we used live-cell reporters for CDK4/6, CDK2, and APC/C^Cdh1^ activities^24,25,32–34^ in non-transformed mammary epithelial MCF-10A cells (Fig. 1A). Of note, the CDK2 reporter measures the collective activity of cyclin E/A-CDK1/2 complexes^35^. To correct for the CDK1/2 contribution in CDK4/6 reporter measurements^24,36^, we also applied the correction factor described previously (see “Methods”)^24^. In the same cells, we performed immunostaining to measure Rb phosphorylation at S807/811 and mRNA fluorescence in situ hybridization (FISH) to measure the E2F target gene, E2F1. Rb phosphorylation at S807/811 is correlated with E2F activity in cycling cells^21,25,37^ and is proposed as a marker for Rb hyperphosphorylation^21^. To investigate the sequence of events in cell-cycle entry, we synchronized cells in quiescence (G0) by mitogen starvation for 48 h, then stimulated the cells with mitogens and fixed them at various time points. Using CDK1 siRNA, we first checked that CDK1 knockdown did not delay activation of the CDK2 reporter in CDK4/6 activating cells (Fig. S1). Given CDK1 is an E2F target gene^38^ and cyclin E prefers CDK2 over CDK1^39^, these data indicate that the CDK2 reporter primarily measures CDK2 activity in our experimental condition. Based on the distribution of activities and Rb phosphorylation in cells stimulated with mitogens for 14 h, we set thresholds to classify populations for active CDK4/6 and CDK2, inactive APC/C^Cdh1^, and Rb phosphorylation at S807/811 (Fig. 1B). We plotted the percentage of cells in each population with averaged E2F1 mRNA levels as a function of time after mitogen stimulation. Our data show that CDK4/6 activation, Rb phosphorylation at S807/811, and activity of E2F transcription were tightly coordinated before CDK2 activation and APC/C^Cdh1^ inactivation (Fig. 1C). Furthermore, at the single-cell level, CDK4/6 activation and Rb phosphorylation at S807/811 were strongly correlated, while CDK2 activity started increasing only after Rb phosphorylation at S807/811 (Fig. 1D). By analyzing three-dimensional activity maps of CDK4/6 versus CDK2 where the percentage of inactive APC/C^Cdh1^ and Rb phosphorylation at S807/811 and E2F1 mRNA levels are color-coded, we found that activation of CDK4/6 tightly correlated with Rb phosphorylation at S807/811 and increased CDK2 activity and E2F1 mRNA levels (Fig. 1E). APC/C^Cdh1^ was inactivated only in the cells with both CDK4/6 and CDK2 activation. Using multiplex staining^40^, we further found that CDK4/6 activity closely associated with multiple sites of Rb phosphorylation at S807/811, T373, S608, and S780 (Fig. 1F,G). Our data indicate that activation of CDK4/6 is tightly coupled with multiple Rb phosphorylation sites and with the initiation of CDK2 and E2F activities.

**Figure 2:**
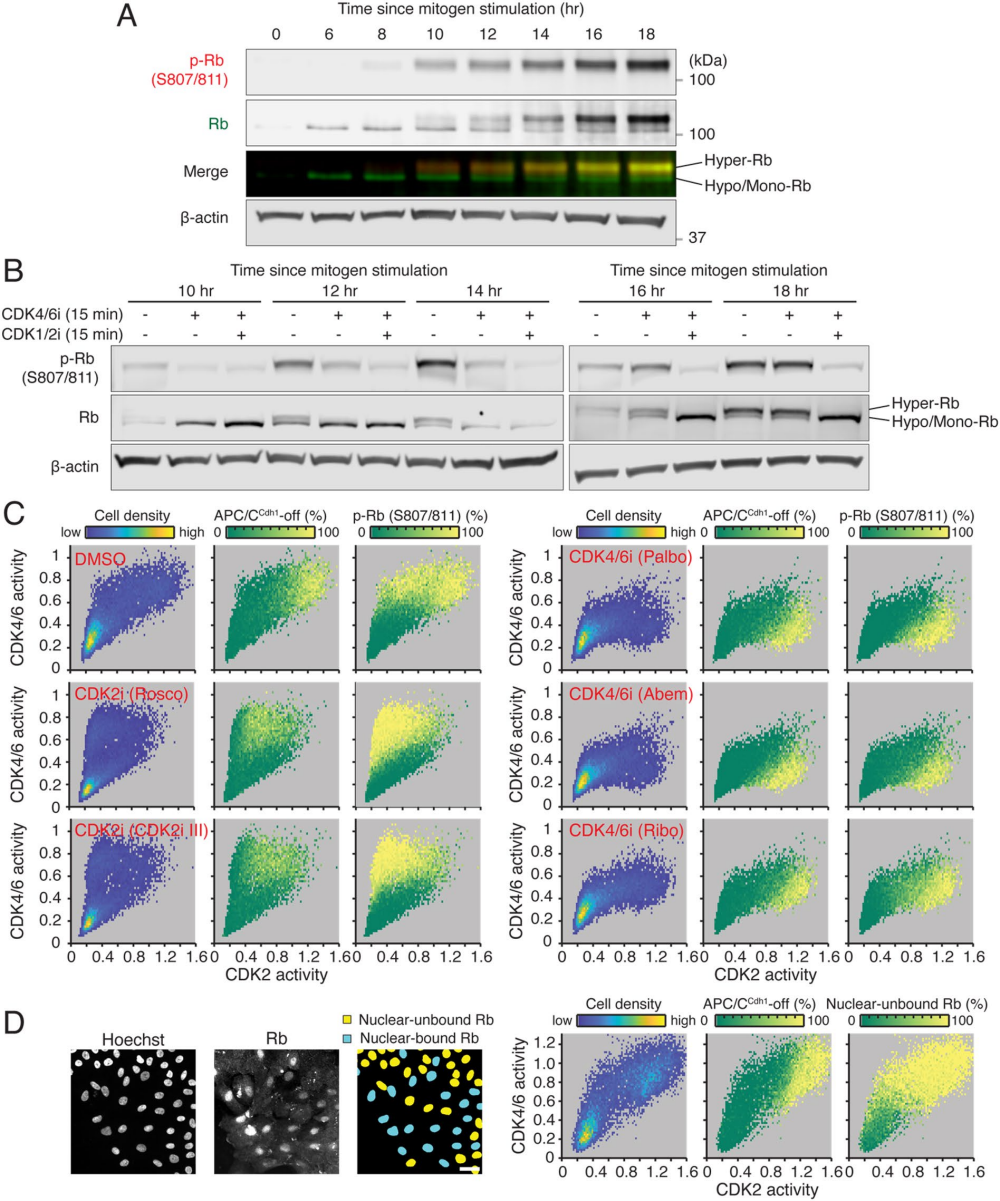
CDK4/6 activation induces Rb hyperphosphorylation and inactivation. (**A**,**B**) Immunoblot of p-Rb (S807/811), total Rb, and β-actin in MCF-10A cells at various time points after mitogen stimulation. After 48 h mitogen removal, MCF-10A cells were stimulated with mitogens for indicated time and then treated with DMSO, palbociclib (1 µM), or palbociclib (1 µM) + CDK1/2 inhibitor III (1 µM) for 15 min prior to harvesting cells (**B**). (**C**) Three-dimensional activity maps of CDK4/6 versus CDK2 activity where cell density, the percentage of inactivated APC/C^Cdh1^, and the percentage of p-Rb at sites S807/811 are color-coded. After 48 h mitogen removal, MCF-10A cells were stimulated with mitogens for 12 h and treated with DMSO, roscovitine (60 µM), CDK2 inhibitor III (60 µM), palbociclib (1 µM), abemaciclib (1 µM), or ribociclib (1 µM) for 1 h prior to fixation. (**D**) Left, representative images of nuclear-bound Rb after in situ extraction and classification of nuclear-bound and nuclear-unbound Rb. Scale bar is 20 μm. Right, three-dimensional activity maps of CDK4/6 versus CDK2 with corresponding cell density, the percentage of inactivated APC/C^Cdh1^, and the percentage of nuclear-unbound Rb color-coded (left to right). After 48 h mitogen removal, MCF-10A cells were stimulated with mitogens for 12 h prior to in situ extraction and fixation.

To evaluate our single-cell data, we next performed immunoblotting and classified hyperphosphorylated and hypo/monophosphorylated Rb. Consistent with our single-cell analysis, we observed Rb hyperphosphorylation starting from 8 to 10 h after mitogen stimulation (Fig. 2A). Using the CDK4/6 inhibitor palbociclib, we tested when Rb hyperphosphorylation become resistant to acute CDK4/6 inhibition for 15 min. We found that palbociclib treatment alone near completely suppressed Rb hyperphosphorylation in cells stimulated with mitogens for 10, 12, and 14 h (Fig. 2B). However, after 16 h of mitogen stimulation, when most cells activate CDK2 based on our single-cell data (Fig. 1C), Rb hyperphosphorylation was resistant to palbociclib treatment alone, but was reversed by co-treatment with palbociclib and CDK1/2 inhibitor (CDK1/2 inhibitor III) (Fig. 2B). We next analyzed three-dimensional activity maps of CDK4/6 versus CDK2, in which the percentage of inactive APC/C^Cdh1^ and Rb phosphorylation are color-coded. Our data showed that all three CDK4/6 inhibitors (palbociclib, abemaciclib, and ribociclib) reversed Rb phosphorylation at S807/811, T373, S608, and S780, while none of the sites were inhibited by treatment with CDK2 inhibitors (roscovitine and CDK2 inhibitor III) (Fig. 2C and Fig. S2A–C). We note that Rb phosphorylation was resistant to CDK4/6 inhibition in cells with high levels of CDK2 activity. The phosphorylated, inactive form of Rb has previously been shown to be associated with reduced binding to the nuclear compartment^21,41^. We further used in situ extraction of soluble proteins with a low-salt buffer in MCF-10A cells to induce the loss of inactivated Rb and the retention of nuclear-bound Rb, inferring the active Rb state. Consistent with the relationship between CDK4/6 activity and Rb phosphorylation, CDK4/6 activity was inversely correlated with nuclear-bound Rb (Fig. S2D). We classified cells into nuclear-bound Rb and nuclear-unbound Rb populations and analyzed three-dimensional activity maps of CDK4/6 versus CDK2 where the percentage of nuclear-unbound Rb is color-coded. Our single-cell data reveal that CDK4/6 activation is tightly coupled with nuclear-unbound Rb before CDK2 activation (Fig. 2D). Together, our data suggest that CDK4/6 activity is sufficient to trigger Rb inactivation.

### Rb phosphorylation by CDK4/6 is sufficient to initiate E2F activity

**Figure 3:**
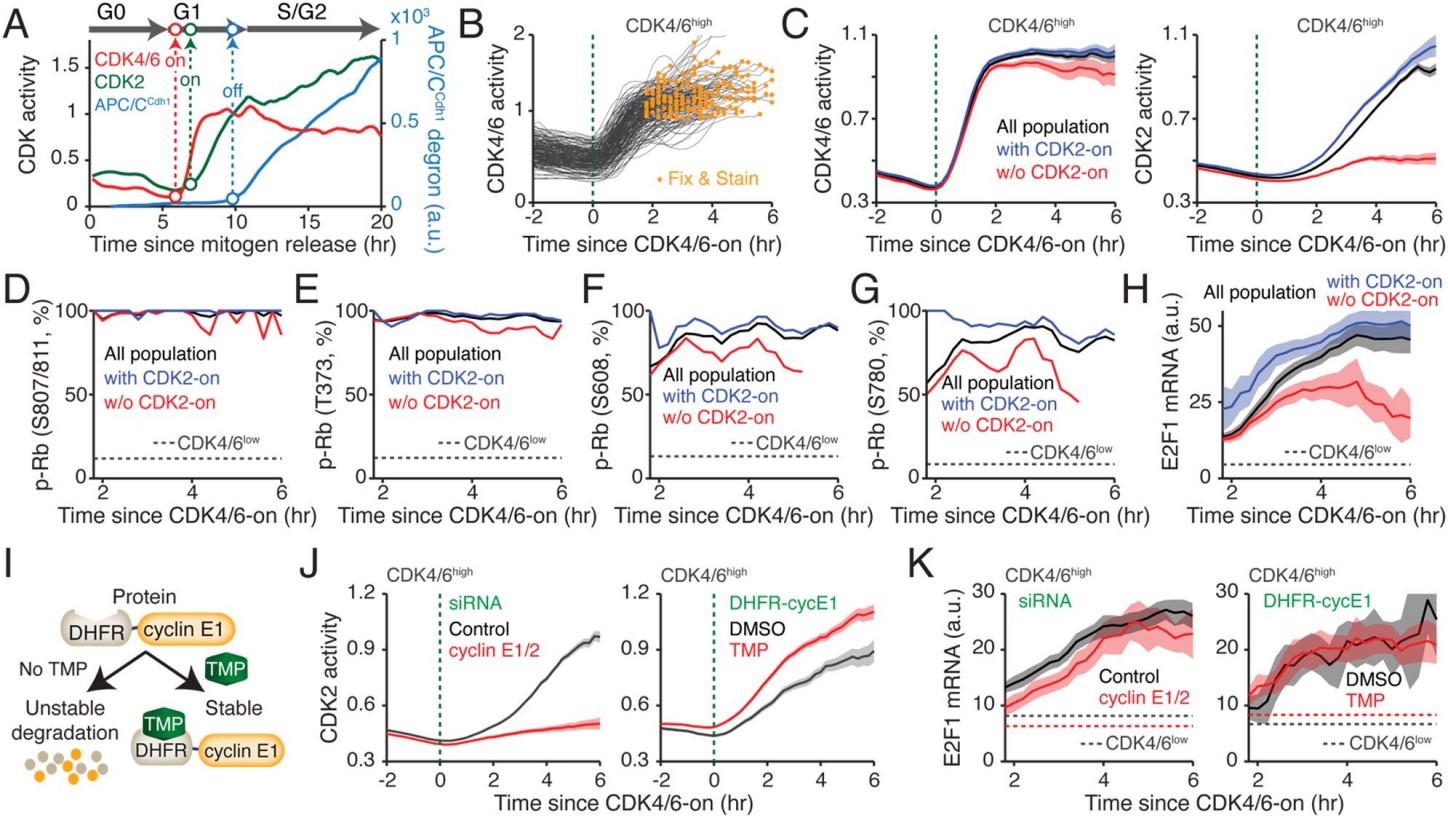
CDK4/6 activation triggers Rb phosphorylation and E2F activation regardless of CDK2 activation kinetics. (**A**) Representative time course analysis of CDK4/6 and CDK2 activities and APC/C^Cdh1^ degron intensity. Cell-cycle phase and point of CDK activation and APC/C^Cdh1^ inactivation are labeled. (**B**) Single-cell traces of CDK4/6 activity aligned to the time of CDK4/6 activation in CDK4/6^high^ cells. To create more heterogeneity, mitogen-starved MCF-10A cells were stimulated with mitogens for various durations (9, 10, and 11 h) prior to fixation. Orange dots correspond to the fixation point. Green dotted line indicates CDK4/6 activity onset. (**C**) Averaged CDK4/6 activity traces (left) and averaged CDK2 activity traces (right) aligned to CDK4/6 activation in MCF-10A cells. Black, blue, and red lines correspond to all population, cells with CDK2 activity, and cells without CDK2 activity, respectively. Green dotted line indicates CDK4/6 activity onset. Data are mean ± 95% confidence interval (All population, *n* = 1,917 cells; with CDK2 activity, *n* = 879 cells; w/o CDK2 activity, *n* = 1,038 cells). (**D**‒**G**) Percentage of p-Rb at sites S807/811 (**D**), T373 (**E**), S608 (**F**), and S780 (**G**), as a function of CDK4/6 activation in MCF-10A cells. Dotted line indicates percentage of p-Rb in CDK4/6^low^ cells. Black, blue, and red lines correspond to all cells, cells with CDK2 activity, and cells without CDK2 activity, respectively. (**D**) All population, *n* = 1,917 cells; with CDK2 activity, *n* = 1,038 cells; w/o CDK2 activity, *n* = 879 cells; CDK4/6^low^ cells, *n* = 4,127 cells. (**E**) All population, *n* = 825 cells; with CDK2 activity, *n* = 545 cells; w/o CDK2 activity, *n* = 280 cells; CDK4/6^low^ cells, *n* = 2,333 cells. (**F**,**G**) All population, *n* = 494 cells; with CDK2 activity, *n* = 332 cells; w/o CDK2 activity, *n* = 162 cells; CDK4/6^low^ cells, *n* = 1,253 cells. (**H**) Averaged E2F1 mRNA levels aligned to the time of CDK4/6 activation. Black, blue, and red lines correspond to all population, cells with CDK2 activity, and cells without CDK2 activity, respectively. Dotted line indicates averaged mRNA level in CDK4/6^low^ cells. Data are mean ± 95% confidence interval (All population, *n* = 1,917 cells; with CDK2 activity, *n* = 1,038 cells; w/o CDK2 activity, *n* = 879 cells; CDK4/6^low^ cells, *n* = 4,493 cells). (**I**) Schematic of TMP-induced rapid expression of cyclin E1. (**J**) Averaged CDK2 activity traces aligned to the time of CDK4/6 activation in CDK4/6^high^ cells. Green dotted line indicates CDK4/6 activity onset. Data are mean ± 95% confidence interval (siRNA: Control, *n* = 3,172 cells; cyclin E1/2, *n* = 1,042 cells; DHFR-cyclin E1: DMSO, *n* = 470 cells; TMP, *n* = 932 cells). (**K**) Averaged E2F1 mRNA levels aligned to the time of CDK4/6 activation in CDK4/6^high^ cells. Dotted lines indicate the averaged E2F1 mRNA level in CDK4/6^low^ cells for each treatment. Data are mean ± 95% confidence interval (siRNA: Control, *n* = 1,699 cells; cyclin E1/2, *n* = 644 cells; DHFR-cyclin E1: DMSO, *n* = 233 cells; TMP, *n* = 552 cells). (**J**,**K**) Left, mitogen-starved MCF-10A cells transfected with either control (black) or cyclin E1/2 siRNA (red) were stimulated with mitogens for various durations (9, 10, and 11 h) prior to fixation. Right, mitogen-starved MCF-10A cells expressing DHFR-cyclin E1 were stimulated with mitogens for various durations (9, 10, and 11 h) together with either DMSO (black) or TMP (50 µM, red) prior to fixation.

To explore the kinetics of Rb phosphorylation and E2F activity regarding CDK4/6 and CDK2 activation, we performed live-cell imaging to monitor CDK4/6, CDK2, and APC/C^Cdh1^ activities and measured Rb phosphorylation and mRNA levels of E2F target genes in MCF-10A cells by fixed-cell analysis. After mitogen stimulation, mitogen-starved cells either activated CDK4/6 to initiate proliferation (CDK4/6^high^) or maintained low CDK4/6 activity to remain in quiescence (CDK4/6^low^) (Fig. S3A)^24^. CDK4/6^high^ cells consequently induced CDK2 activation and APC/C^Cdh1^ inactivation and exhibited substantial heterogeneity in the timing of CDK4/6 activation, allowing us to measure Rb phosphorylation and E2F target genes at various time points after CDK4/6 activation (Fig. 3A and Fig. S3A). By mapping fixed-cell data back to live-cell data at the single-cell level, we compared Rb phosphorylation levels between CDK4/6^high^ and CDK4/6^low^ cells and found that all five Rb phosphorylation sites were highly correlated with CDK4/6^high^ cells (Fig. S3B).

When we aligned live-cell traces and fixed data by the time of CDK4/6 activation (CDK4/6-on), we found heterogeneous responses in the timing of CDK2 activation (CDK2-on) (Fig. 3B and Fig. S3C). Approximately 40% of CDK4/6^high^ cells did not activate CDK2 before fixation (Fig. S3D). To test how CDK2 activation influences the kinetics of Rb phosphorylation, we further classified CDK4/6^high^ cells into populations with or without CDK2 activation (Fig. 3C and Fig. S3E–G). When we plotted the percentage of Rb phosphorylation after CDK4/6 activation, we found Rb phosphorylation at S807/811, T373, S608, and S780 in CDK4/6^high^ cells regardless of CDK2 activity, indicating that CDK4/6 activation alone is capable of inducing multiple sites of Rb phosphorylation (Fig. 3D–G). However, when we measured E2F activity, CDK4/6^high^ cells with CDK2 activation had higher levels of E2F-target genes, E2F1, Cdc25A, and cyclin E2 mRNA, and cyclin E2 protein (Fig. 3H and Fig. S4). We next tested whether CDK2 activity facilitates E2F activation kinetics, or whether E2F activity levels correlate with the classification of CDK4/6^high^ cells by manipulating CDK2 activity and measuring the kinetics of E2F activity. To modulate CDK2 activity, we used cyclin E1/2 siRNA and the dihydrofolate reductase (DHFR)-trimethoprim (TMP)-cyclin E1 protein stabilization system^42^, which decreases and increases cyclin E expression, respectively. In the absence of TMP, cyclin E1 protein conjugated with the DHFR domain is continuously degraded by the proteasome (Fig. 3I). Addition of TMP rapidly stabilizes and thereby increases exogenous cyclin E1 protein. Knockdown of cyclin E1/2 significantly reduced expression levels of cyclin E (Fig. S5A). In addition, our data show that knockdown of cyclin E1/2 and overexpression of cyclin E1 in CDK4/6^high^ cells delayed and accelerated CDK2 activation, respectively, without promoting CDK4/6 activation (Fig. 3J, Fig. S5B,C). However, mRNA expression kinetics of E2F1 and Cdc25A after CDK4/6 activation were similar in CDK4/6^high^ cells regardless of CDK2 activity (Fig. 3K, Fig. S5D,E). We also found that knockdown of CDK2 did not reduce kinetics of E2F1 mRNA transcription after CDK4/6 activation (Fig. S5F–H). Taken together, the kinetics of Rb phosphorylation and mRNA levels of E2F target genes suggest that Rb phosphorylation by CDK4/6 activity is sufficient to initiate E2F induction regardless of CDK2 activation kinetics.

### High CDK2 activity is required to initiate CDK4/6-independent cell-cycle progression

**Figure 4:**
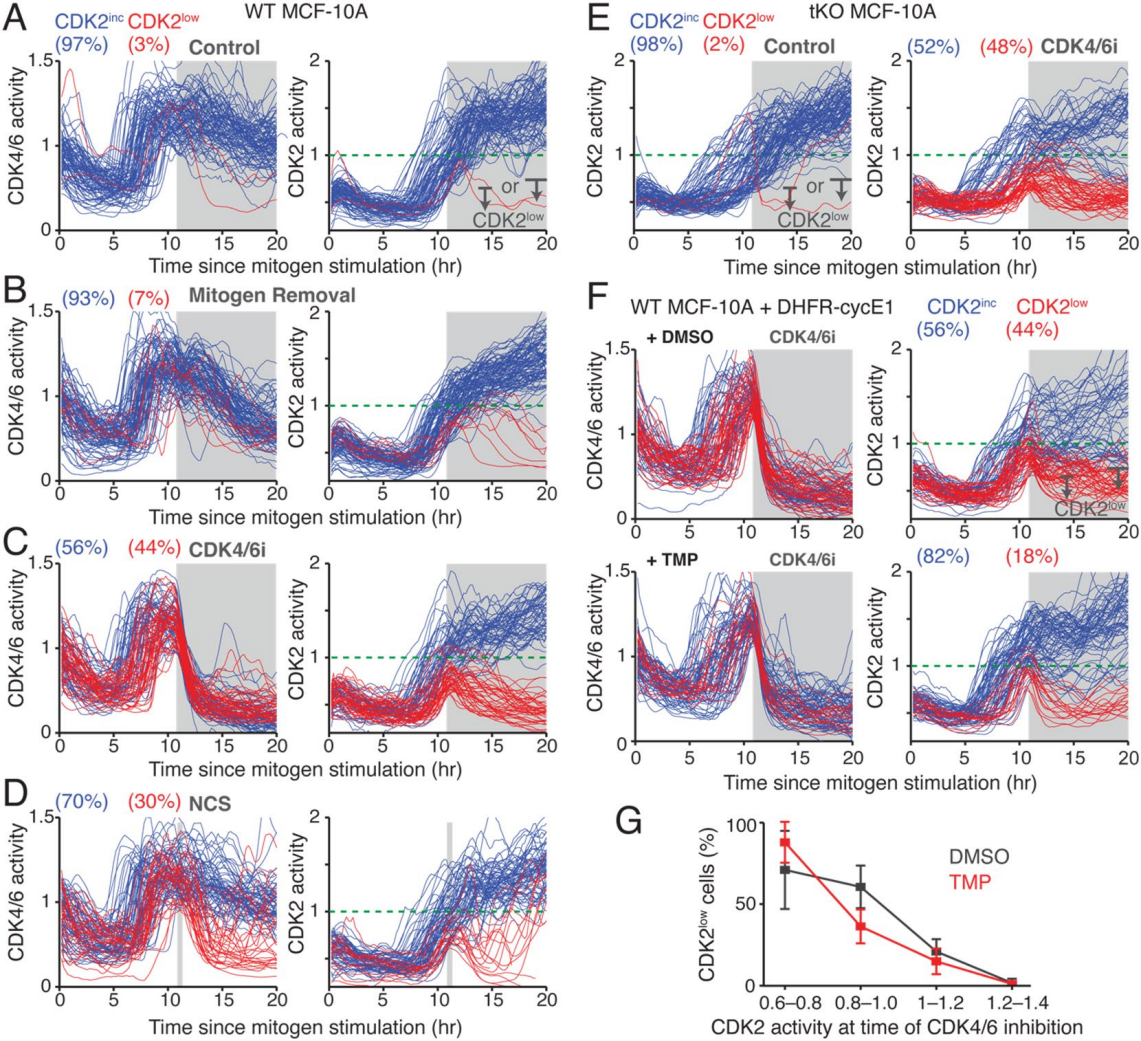
High CDK2 activity is required to initiate CDK4/6-independent cell cycle progression. (**A**‒**D**) Single-cell traces of CDK4/6 activity (left) and CDK2 activity (right) in wild type MCF-10A cells. Top to bottom, corresponding conditions under control (**A**), mitogen withdrawal (**B**), palbociclib (1 µM) (**C**), and 10 min NCS pulse (100 ng/ml) (**D**). All treatments performed 11 h after mitogen stimulation (marked in gray). Blue and red lines correspond to CDK2^inc^ and CDK2^low^ cells, respectively. Cells were classified based on CDK2 activity using 2 windows as indicated in (**A**). (**E**) Single-cell traces of CDK2 activity in p21/p27/p57 tKO MCF-10A cells. Control (left) and palbociclib (1 µM) (right) treatments were performed 11 h after mitogen stimulation (marked in gray). (**F**,**G**) Single-cell traces of CDK4/6 activity (left) and CDK2 activity (right) (**F**). Percentages of CDK2^low^ cells increasing CDK2 activity at the time of inhibition (**G**). After 48 h mitogen removal, MCF-10A cells expressing DHFR-cyclin E1 were stimulated with mitogens together with DMSO or TMP (50 µM). Palbociclib (1 µM) treatment were performed 11 h after mitogen stimulation. Data are mean ± s.d. (*n* = 5 biological replicates) (**G**).

We next evaluated when cells trigger CDK4/6-independent cell-cycle progression. To inhibit CDK4/6 activity, we applied palbociclib, induced stress signaling, or removed mitogens 11 h after mitogen stimulation. Previous studies showed that induction of stress signaling rapidly inactivates CDK4/6 while mitogen removal slowly inactivates it^21,24^. To induce stress signaling, we used a pulse of the DNA damage reagent, neocarzinostatin (NCS) for 10 min, which produces exogenous DNA double-stranded breaks. For analysis, we selected CDK4/6^high^ cells with CDK2 activation (> 0.6) at the time of inhibition. Based on CDK2 response to the treatment, we used two windows to further classify cells by path, either continuing to increase CDK2 activity for proliferation (CDK2^inc^) or reversing CDK2 activation and entering quiescence (CDK2^low^) (Fig. 4A). A recent report suggested that mitogen removal creates transient hysteresis in CDK4/6 activity^21^. Consistently, mitogen removal inactivated CDK4/6 with a 4–5-h delay and slightly increased the percentage of CDK2^low^ cells (Fig. 4B). However, rapid CDK4/6 inactivation by palbociclib and NCS pulse^24^ suppressed both CDK4/6 and CDK2 activities (Fig. 4C,D). When CDK2 activity was approximately over 1 at the time of CDK4/6 inhibition, cells tended to continue through cell-cycle entry regardless of CDK4/6 activity. Furthermore, we found that while treatment with palbociclib still induced cell-cycle exit in p21 knockout cells with low CDK2 activity, the effects of NCS were absent in p21 knockout cells (Fig. S6A). This suggests that the p53/p21 pathway is responsible for DNA damage-mediated CDK4/6 inactivation, consistent with a previous study showing that the ratio between nuclear cyclin D1 and p21 levels determines CDK4/6 activity and cell-cycle entry in the G1 phase^37^.

We next tested whether redistribution of Cip/Kip family proteins promotes inactivation of low CDK2 activity and cell-cycle exit by palbociclib treatment. To test this, we used CRISPR-Cas9 system to establish p21, p27, and p57 tKO MCF-10A cells (Fig. S6B). We found that palbociclib treatment still induced cell-cycle exit in tKO MCF-10A cells with low CDK2 activity, indicating that CDK4/6 inhibition leads to Rb activation and inactivation of low CDK2 activity (Fig. 4E). Considering possible contribution of CDK1 activity to the CDK2 reporter^35^, we knocked down CDK1 and found no increases in CDK2^low^ cells after CDK4/6 inhibition, suggesting that CDK2 is primarily responsible for the initiation of CDK4/6-independent cell-cycle entry (Fig. S6C).

Previous studies showed that CDK2 activation by cyclin E overexpression can bypass CDK4/6 activity^19,43–46^. Thus, we hypothesize that while cyclin E overexpression increases CDK2 activity and the percentage of cells initiating CDK4/6-independent cell-cycle progression, CDK2 activity at the time of CDK4/6 inhibition predicts cell-cycle fate regardless of cyclin E overexpression. We used the DHFR-cyclin E1 protein stabilization system to increase CDK2 activity. We added palbociclib 11 h after mitogen stimulation with or without TMP, and selected CDK4/6^high^ cells with CDK2 activation (> 0.6). While we confirmed that increased CDK2 activity led to a higher percentage of CDK2^inc^ cells, our data showed that CDK2 activity at the time of CDK4/6 inhibition equally predicted cell-cycle entry or exit despite cyclin E1 overexpression (Fig. 4F,G, and Fig. S6D). Together our data suggest that high CDK2 activity is required to initiate CDK2-Rb positive feedback and CDK4/6-independent cell-cycle progression.

### High CDK2 activity coordinates the timing of CDK2-Rb feedback and sequential DNA replication

**Figure 5:**
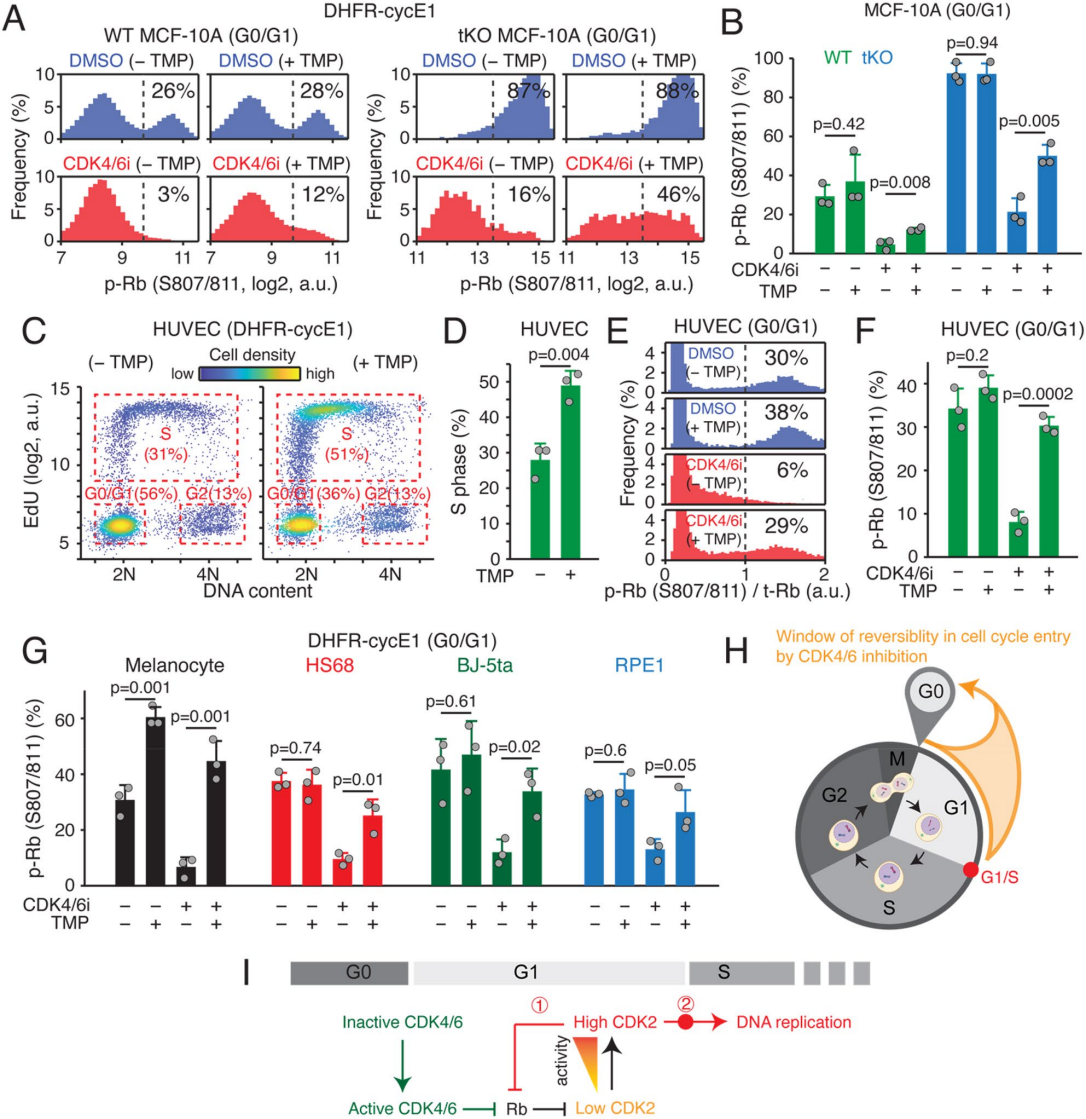
CDK2 activity regulates the timing of CDK2-Rb feedback and the G1/S transition. (**A**,**B**) Histogram (**A**) and the percentage (**B**) of p-Rb at S807/811 in wild type and p21/p27/p57 tKO MCF-10A cells with DHFR-cyclin E1 expression in G0/G1 phase. After 48 h mitogen removal, cells were stimulated with mitogens for 11 h with or without TMP (50 µM) followed by acute treatment with EdU (10 µM) ± palbociclib (1 µM) for 15 min prior to fixation. Data are mean ± s.d. (*n* = 3 biological replicates) (**B**). *P*-values were calculated with two-sample *t*-tests. (**C**,**D**) Scatterplot of Hoechst versus EdU, where cell density is color-coded (**C**). The percentage of S phase cells (**D**). Cycling HUVECs expressing DHFR-cyclin E1 were treated with or without TMP (50 µM) for 6 h followed by EdU (10 µM) treatment for 15 min prior to fixation. Data are mean ± s.d. (*n* = 3 biological replicates) (**D**). *P*-values were calculated with two-sample *t*-tests. (**E**) Histogram of p-Rb at S807/811 in cycling HUVEC cells expressing DHFR-cyclin E1 in G0/G1 phase. Cycling cells were treated with or without TMP (50 µM) for 6 h followed by acute treatment with palbociclib (1 µM) and EdU (10 µM) for 15 min prior to fixation. (**F**,**G**) Percentage of p-Rb at S807/811 for each treatment condition as indicated in HUVEC (**F**), Melanocyte, HS68, BJ-5ta, and RPE1 cells (**G**) expressing DHFR-cyclin E1 in G0/G1 phase. Data are mean ± s.d. (*n* = 3 biological replicates). Cycling cells were treated with or without TMP (50 µM) for 6 h followed by treatment with palbociclib (1 µM) and EdU (10 µM) for 15 min prior to fixation. *P*-values were calculated with two-sample *t*-tests. (**H**,**I**) Schematic of sequential regulation of the Rb/E2F pathway by CDK4/6 and CDK2. CDK4/6 inactivates Rb to induce E2F activity and a gradual increase in CDK2 activity. High levels of CDK2 activation triggers CDK2-Rb feedback to commit to the cell cycle ①, then initiates DNA replication ②.

We next sought to determine whether high cyclin E-CDK2 activity is sufficient, or if additional mechanisms in S phase are required to trigger CDK2-Rb positive feedback. To address this, we tested whether cyclin E1 overexpression is enough to induce CDK4/6-independent Rb phosphorylation before DNA replication. We used the DHFR-cyclin E1 protein stabilization system to acutely increase CDK2 activity. To exclude the possibility of contribution by other regulatory mechanisms during S phase, we used 5-ethynyl-20-deoxyuridine (EdU) and Hoechst staining to gate cells in G0/G1. In addition, we treated both wild-type and tKO MCF-10A cells with palbociclib for 15 min to ensure the effect is not due to the indirect inhibition of CDK2 by Cip/Kip family protein redistribution^30^. After mitogen starvation for 48 h, we stimulated cells with mitogens for 11 h prior to palbociclib treatment. During G0/G1, while control cells dramatically lost Rb phosphorylation at S807/811 following acute CDK4/6 inhibition for 15 min, an increase in CDK2 activity by cyclin E1 overexpression significantly increased the percentage of Rb phosphorylation at S807/811 in both wild-type and tKO MCF-10A cells (Fig. 5A,B, and Fig. S7A). We also found that cyclin E1 overexpression rescued inhibition of other Rb phosphorylation sites at T373 and S608, following palbociclib treatment in tKO MCF-10A cells (Fig. S7B–E). In addition, we observed that an increase in CDK2 activity accelerated S-phase entry (Fig. S7F). This is consistent with previous reports showing that CDK2 activity controls not only Rb phosphorylation, but also the initiation and progression of DNA replication^47–49^. We next tested the effect of cyclin E1 overexpression on initiation of CDK2-Rb feedback and DNA replication in asynchronously cycling cells. When we increased CDK2 activity by addition of TMP in passage-limited human umbilical vein endothelial cells (HUVECs) expressing DHFR-cyclin E1 for 6 h, the percentage of S-phase cells was significantly increased (Fig. 5C,D), confirming that CDK2 activity facilitates S-phase entry in cycling cells. In addition, while we consistently found loss of Rb phosphorylation at S807/811 in G0/G1 by acute CDK4/6 inhibition, an increase in CDK2 activity significantly rescued the reduction of Rb phosphorylation at S807/811 (Fig. 5E,F). These results suggest that high cyclin E1-CDK2 activity is sufficient to trigger Rb phosphorylation in G1 phase. We observed similar results that CDK2 sequentially regulates the timing of CDK2-Rb feedback and S-phase entry in passage-limited human melanocytes, and fibroblasts HS68, as well as hTERT-immortalized fibroblasts BJ-5ta and retinal pigment epithelial RPE1 cells (Fig. 5G and Fig. S7G). These data indicate that regulation of Rb phosphorylation and DNA replication by high cyclin E1-CDK2 activity is conserved across primary and immortalized cell types. Taken together, our data suggest that high CDK2 activity coordinates the timing of CDK2-Rb feedback and the G1/S transition.

## Discussion

Our studies show that CDK4/6 activity initiates E2F activation, and CDK2 activity coordinates the timing of CDK2-Rb feedback and DNA replication in both mitogen-starved and cycling cells (Fig. 5H,I). Our data indicate that Rb phosphorylation by CDK4/6 activity is tightly correlated with the initiation of E2F transcription activity. Furthermore, by monitoring the kinetics of E2F activity and manipulating CDK2 activity, our data reveal that active CDK4/6 can trigger E2F activity regardless of CDK2 activation kinetics, suggesting that Rb phosphorylation by CDK4/6 activity is sufficient to trigger E2F activation. In addition, our study shows that high CDK2 activity is required to initiate CDK2-Rb positive feedback and consequently CDK4/6-indendent cell-cycle progression before promoting DNA replication.

A recent study demonstrated that Rb phosphorylation and cell-cycle entry can be reversed by acute palbociclib treatment until the onset of S-phase entry^21^, challenging the notion that cyclin E-CDK2 phosphorylates Rb in cells. It was also unclear whether reversibility of Rb phosphorylation and cell-cycle entry by CDK4/6 inhibitors is due to redistribution of Cip/Kip family proteins to CDK2 activity^30,31^. Using p21/p27/p57 tKO cells, our data suggest that on-target effects of CDK4/6 inhibitors may reverse Rb phosphorylation and cell-cycle entry in cells with low CDK2 activity. Additionally, alternative rapid CDK4/6 inhibition by a NCS pulse further supports that high CDK2 activity is required to initiate CDK4/6-independent cell-cycle progression. Recent studies demonstrated that combined CDK2 knockout and treatment with CDK4/6 inhibitors synergistically suppressed cell proliferation in lung and breast cancer, supporting on-target effect of CDK4/6 inhibitors^50,51^. By inducing cyclin E1 overexpression, we show that high cyclin E1-CDK2 activity is required to initiate CDK2-Rb positive feedback, reconciling previous reports and recent live-cell experiments with CDK4/6 inhibitors. Regarding CDK2 knockout cells^52,53^, it has been reported that CDK1 binds to cyclin E and compensates for the loss of CDK2 by phosphorylating CDK2 substrates^54^. Notably, in the absence of CDK2/4/6, CDK1 binds to all cyclins and is sufficient to trigger cell-cycle entry^55^.

Our study focused on examining CDK2-Rb positive feedback in regard to CDK4/6-independent cell-cycle progression during G1 and S phases. Other regulatory components downstream of Rb phosphorylation, such as positive feedback from Skp2 autoinduction^56–59^ or E2F autoregulation^60,61^ and double negative feedback between Emi1 and APC/C^Cdh1^^25,26^, have been suggested to regulate irreversible cell-cycle entry. Thus, future studies are needed to understand how different networks cooperatively regulate irreversible cell-cycle entry. We also note limitation of our approach in differentiating between mono- and hyper-phosphorylation of Rb. Additionally, using in vitro kinase assay experiments, a previous study demonstrated CDK4/6 activation 1 h after mitogen stimulation and in contact-inhibited condition^22^. However, using the CDK4/6 reporter, our single-cell data showed that subsets of cells started activating CDK4/6 3 h after mitogen stimulation (Fig. S3A), and contact inhibition suppressed CDK4/6 activity (Fig. S8). The differences in kinetics of CDK4/6 activity from these assays might be possibly due to the variation in sensitivity of different assays or from measuring isolated versus intact cyclin D-CDK4/6 complexes. As we previously described^24^, the delayed CDK4/6 activation could be also explained by stoichiometric inhibition of cyclin D-CDK4/6 complexes by non-phosphorylated Cip/Kip member proteins^30,37,62–64^. Given p27 induction by contact inhibition^62,65^, non-phosphorylated p27 may explain CDK4/6 inhibition in contact-inhibited condition. Consistently, cyclin D1 levels alone are not correlated with cell-cycle entry in vitro^37,66^ and therapeutic outcomes of CDK4/6 inhibitors in breast cancer patients^67,68^.

Recent other studies indicate that mitogen removal and various forms of stress induction respectively cause slow and rapid inactivation of CDK4/6^21,24^. Depending on the strength of anti-mitogens, different external stimuli may reverse CDK4/6 activation with varying kinetics. Consequently, individual external stimuli could reverse Rb phosphorylation and cell-cycle entry at different time points before high CDK2 activation. In the presented study, using CDK2 and CDK4/6 sensors and single-cell imaging approaches, we demonstrated that CDK4/6 phosphorylates Rb to initiate E2F and CDK2 activity and that high CDK2 activity sequentially triggers CDK2-Rb feedback and the G1/S transition. Our findings provide insight into a mechanism for cell-cycle entry depending on external conditions to control cell-cycle commitment and the G1/S transition.

## Methods

### Cell culture

MCF-10A human mammary epithelial cells (ATCC, CRL-10317) were cultured in phenol red-free DMEM/F12 (Invitrogen) and supplemented with 5% horse serum, 20 µg/ml EGF, 10 µg/ml insulin, 500 µg/ml hydrocortisone, 100 ng/ml cholera toxin, 50 U/ml penicillin, and 50 µg/ml streptomycin. For mitogen starvation, DMEM/F12 plus 0.3% BSA w/v, 500 µg/ml hydrocortisone, and 100 ng/ml cholera toxin was used. Human primary epidermal melanocytes (ATCC, PCS-200-013) were culture in Dermal Cell Basal Medium (PCS-200-030). HS68 primary human foreskin fibroblasts (ATCC, CRL-1635) were cultured in DMEM (Invitrogen) plus 10% FBS, 50 U/ml penicillin, and 50 µg/ml streptomycin. HUVECs human umbilical vein endothelial cells (Lonza, C2519A) were cultured in Endothelial Cell Growth Medium (Promo Cell, C-22010). BJ-5ta human foreskin fibroblasts (ATCC, CRL-4001) were cultured in DMEM (Invitrogen) plus 10% FBS, 50 U/ml penicillin, and 50 µg/ml streptomycin. RPE1-hTERT human retinal pigment epithelial cells (ATCC, CRL-4000) were cultured in DMEM: F12 (Invitrogen) plus 10% FBS and 0.01 mg/ml hygromycin B. All cell lines tested negative for mycoplasma.

### Antibodies and reagents

Palbociclib (S1116), Ribociclib (S7440), Abemaciclib (S7158) and Roscovitine (S1153) were purchased from Selleck Chemicals. Neocarzinostatin (N9162) was obtained from Sigma-Aldrich. CDK2 inhibitor III (238803) and CDK1/2 inhibitor III (217714) were purchased from EMD Millipore Sigma. Trimethoprim (TMP; 16473) was obtained from Cayman Chemical. Anti-phosphor-Rb (Ser807/811) (8516), anti-phosphor-Rb (S608) (2181), anti-p57 (2557) and anti-β-actin (3700) were purchased from Cell Signaling Technology. Anti-phosphor-Rb (S780) (558385), anti-Rb (554136) and anti-p27 (610241) were purchased from BD Bioscience, and anti-phophor-Rb (T373) (ab52975) and anti-cyclin E2 (ab32103) were obtained from Abcam. Anti-Cyclin E (HE12) (sc-247), and anti-GAPDH (FL-335) (sc-25778) were obtained from Santa Cruz.

### Constructs and stable cell lines

pLenti-DHB(a.a.994–1087)-mVenus-p2a-mCherry-Rb (a.a.886–928) and mCerulean-Geminin (a.a.1–110)-p2a-H2B-iRFP670 were described previously^24^. Full sequence and constructs are available from Addgene. DHFR-cyclin E1 was cloned into a pCru5 retroviral vector. To generate stable cell lines, lentiviral and retroviral constructs were introduced into MCF-10A, melanocyte, HS68, BJ-5ta, and RPE1-hTERT cells by viral transduction.

### Knockout cell lines

p21/p27 dKO and p21/p27/p57 tKO MCF-10A cells were generated according to the manufactural guidance (Integrated DNA technologies). Briefly, predesigned crRNA oligos directed at the locus of *CDKN1B* and *CDKN1C* were mixed with tracrRNA oligos tagged fluorescence ATTO 550 in equimolar concentrations to a final duplex concentration of 44 μM and heated at 95 °C for 5 min. Cells were transfected with a gRNA and a Cas9 enzyme. Cells were allowed to recover from the transfection and sorted in single cells by fluorescence-activated cell sorting (FACS). For knockout validation, cells were harvested and immunoblotted by incubating with primary anti-p27 (Cell signaling technology, 3686) and anti-p57 (Cell signaling technology, 2557) and then with anti-rabbit IgG conjugated to horseradish peroxidase (HRP) for chemiluminescent detection.

### Immunofluorescence

Cells were fixed by adding 4% paraformaldehyde at a ratio of 1:1 with culture medium (final 2% paraformaldehyde) for 15 min. Then, cells were washed three times in PBS, followed by incubation in permeabilization/blocking buffer with 0.1% triton X-100, 10% FBS, 1% BSA, and 0.01% NaN_3_ for 1 h, and stained overnight at 4 °C with primary antibodies. Primary antibodies were visualized using a secondary antibody conjugated to Alexa Fluor-488, -568, or -647. For EdU staining, cells were treated with 10 µM EdU for 15 min, then fixed and processed with aziede-modified Alexa Fluor-647 according to manufacturer’s instructions (Invitrogen, #C10269). To prevent the use of fluorophores limited by fluorescent reporters, cells were chemically bleached^69^. Pre-extraction protocol was described previously^70^. Pre-extraction and fixing were performed on an ice block. Iterative indirect immunofluorescence imaging (4i) protocol was described previously^40^.

### RNA FISH

RNA in situ hybridization was carried out using the Affymetrix Quantigene ViewRNA ISH cell assay according to manufacturer’s instructions. Briefly, cells were plated in a 96-well glass plate (Cellvis P96-1.5H-N) that was pre-hybridized with 1:100 collagen (Advanced BioMatrix, #5005-B) in PBS overnight. At the time of fixation, cells were fixed with 4% paraformaldehyde for 15 min and dehydrated overnight using 75% EtOH. After rehydration in PBS for 10 min, cells were permeabilized with 0.2% triton X-100 for 15 min at room temperature, and then treated for probe hybridization, amplification, and labeling with Alexa Fluor-555. Cells were then incubated with Hoechst (1 µg/ml) for 10 min, washed three times with PBS, and left in PBS for imaging. For the cases where immunofluorescence was performed after FISH signal imaging, cells were incubated with the ViewRNA wash buffer to remove the probes and allow for measurement of other fluorophores (Alexa Fluor-488 and Alexa Fluor-647).

### siRNA transfection

siRNA was transfected using DharmaFECT1 Transfection Reagent (Horizon, T-2001) according to the manufacturer’s instructions. The following siRNAs were used as 20 nM concentration: Negative Control, CDK1, CDK2 siRNA (Integrated DNA Technologies [IDT]), CCNE1 and CCNE2 (Dharmacon, siGENOME pool).

### Immunoblot

To measure total and phosphorylated (S807/811) Rb, mitogen-starved MCF-10A cells were stimulated with mitogens and harvested at indicated time points with or without drug treatment for 15 min (Fig. 2A,B). Whole cell lysates were collected using CHAPS lysis buffer containing Halt protease inhibitor cocktail (Thermo Scientific, 78429) and phosphoSTOP (Millipore Sigma, 4906845001). Protein concentrations were measured by Pierce™ 660 nm protein assay reagent (Thermofisher Scientific, 22660) according to manufacturer’s guidance. 30 μg proteins were mixed with LDS sample buffer (Invitrogen, NP0007) containing 2% 2-mercaptoethanol and were incubated at 70 °C for 10 min. Proteins were loaded in Bolt™ 8% Bis–Tris Plus gel (Invitrogen, NW00080) or 6% Tris–Glycine gel. After gel electrophoresis, proteins were transferred to a PVDF membrane by semi-dry transfer (Bio-Rad Trans-Blot Turbo system, 1704150). Membranes were immunoblotted by incubating with primary mouse anti-Rb (4H1) (1:2000; Cell Signaling Technology, 9309), rabbit anti-p-Rb (Ser807/811) (1:1000; Cell Signaling Technology, 8516) and β-actin (1:2000; Cell Signaling Technology, 3700) antibodies at 4 °C overnight, and then with IR Dye 680RD goat anti-rabbit IgG (1:2000; LI-COR, 926-68071) or IgG IR Dye 800CW goat anti-mouse (1:2000; LI-COR, 926-32210) as secondary antibodies for 2 h at room temperature. Blots were visualized by Odyssey CLx Infrared Imaging system (LI-COR). For siRNA validation, cells were collected 48 h after siRNA transfection using CHAPS lysis buffer. Total 40 μg proteins were mixed with LDS sample buffer (Invitrogen, NP0007) and incubated at 70 °C for 10 min. Proteins were loaded on a Bolt™ 4‒12% Bis–Tris Plus gel (Invitrogen, NW04120), and then transferred to a PVDF membrane by a Trans-blot turbo™ transfer system (BioRad) according to the manufacturer’s instruction. Membranes were immunoblotted by incubating with primary mouse anti-Cyclin E (HE12) (1:200; Santa Cruz, sc-247) and rabbit anti-GAPDH (FL-335) (1:2000; Santa Cruz, sc-25778) antibodies at 4 °C overnight, and then with IR Dye 680RD goat anti-rabbit IgG (1:2000; LI-COR, 926-68071) or IgG IR Dye 800CW goat anti-mouse (1:2000; LI-COR, 926-32210) as secondary antibodies for 2 h at room temperature. Blots were visualized by Odyssey CLx Infrared Imaging system (LI-COR). To validate p27 and p57 knockout MCF-10A cells, proteins were resolved on a 4‒12% (Invitrogen, NW04120) and transferred to a PVDF membrane. After incubating in blocking buffer (5% milk-containing TBST) for an hour, membranes were immunoblotted by incubating with rabbit anti-p27 (1:1000; #3868, Cell Signaling Technology), rabbit anti-p57 (1:1000; #2557, Cell Signaling Technology) or rabbit anti-GAPDH (1:2000) and then with goat anti-rabbit IgG conjugated to HRP (1:2000) secondary antibody diluted in blocking buffer. The membranes were then reacted with chemiluminescent substrates (WBLUR0100, Millipore Sigma) and detected using ChemiDoc system (Bio-Rad) recording the protein bands through Image LabTM Software (ver. 5.2.1, Bio-Rad). Full scan images of all immunoblots were shown in Fig. S9. All the blots were cut prior to hybridization with antibodies. Using Odyssey, membrane edges were not visualized.

### Measurement of CDK2 and CDK4/6 activities

The CDK2 reporter generally measures the collective activity of cyclin E/A-CDK1/2 complexes^35^. Since kinase translocation reporters (KTRs) contain a sequence similar to a degenerate CDK2 substrate motif, it is speculated that CDK1/2 may phosphorylate the KTR-based CDK4/6 reporter during S and G2 phases^24,36^. To correct for the CDK2 contribution in CDK4/6 reporter measurements, we applied the correction factor described previously^24^. Briefly, in the presence of CDK4/6 inhibitor, we used linear regression to calculate the contribution of CDK1/2 activities to the CDK4/6 reporter. We subtracted a calculated fraction of the CDK2 reporter signal as shown below.

$${\text{CDK4}}/{6}\;{\text{activity}} = {\text{CDK4}}/{\text{6 reporter}}\;{\text{activity}} - 0.{35} \times {\text{CDK2 reporter}}\;{\text{activity}}$$

### Microscopy

All images were taken on an Axio Observer 7 microscope (Zeiss) using 20X objective (0.8 N.A) with 2-by-2 pixel binning. For live-cell imaging, cells were imaged every 12 min in a humidified and 37 °C chamber in 5% CO_2_. Total light exposure time was kept under 400 ms for each time point.

### Image analysis

All image analyses were performed with custom Matlab scripts as previously described^25,37^. Briefly, after illumination bias correction, cells were segmented, either by using Hoechst staining (fixed-cell imaging) or H2B-iRPF670 (live-cell imaging). For DHB-mVenus and mCherry-Rb measurements, cells were segmented for their cytoplasmic regions by spatially approximating a ring with an inner radius of 2 µm outside of the nuclear mask and an outer radius maximum of 10 µm outside the nuclear mask. Regions within 10 µm of another nucleus were excluded. Regions with pixel intensities indistinguishable from background (discussed below) were also excluded. For RNA FISH measurements, cells were segmented for their whole cell regions using an area that encompasses the nucleus and reaches out as far as 50 µm outside of the nuclear mask while preventing overlap with neighboring cells. A mask of FISH puncta was generated by top hat-filtering raw images with a circular kernel of radius 4 µm and thresholding absolute intensity. The RNA puncta parameter represents an average of the number of pixels in whole cell regions. For cell tracking, the deflection-bridging algorithm was implemented to perform tracking of cells between live-cell frames as well as between the final live-cell frame and subsequent fixed-cell image.

### Statistical analysis

Statistical analyses were based on a two-sample *t*-test. The data were represented either as mean ± s.d. or mean ± 95% confidence intervals and the number of replicates was indicated in respective figure legends. *P*-values were indicated in respective figures. No statistical methods were used to predetermine sample size. The experiments were not randomized, and the investigators were not blinded to allocation during experiments and outcome assessment.

## Supplementary Information

Supplementary Figures.

## Data Availability

The code for the image analysis pipeline is available at https://github.com/scappell/Cell_tracking. Additional modified scripts and datasetsare available upon reasonable request.
