# Supplementary material for: CDK4/6 initiates Rb inactivation and CDK2 activity coordinates cell-cycle commitment and G1/S transition
Source: Sci Rep. 2022 Oct 7;12:16810. doi: 10.1038/s41598-022-20769-5 (PMC9546874; doi:10.1038/s41598-022-20769-5)
Supplement: Supplementary file 1 — Supplementary Figures. [file 41598_2022_20769_MOESM1_ESM.docx]

**Supplemental Figure 1**

**
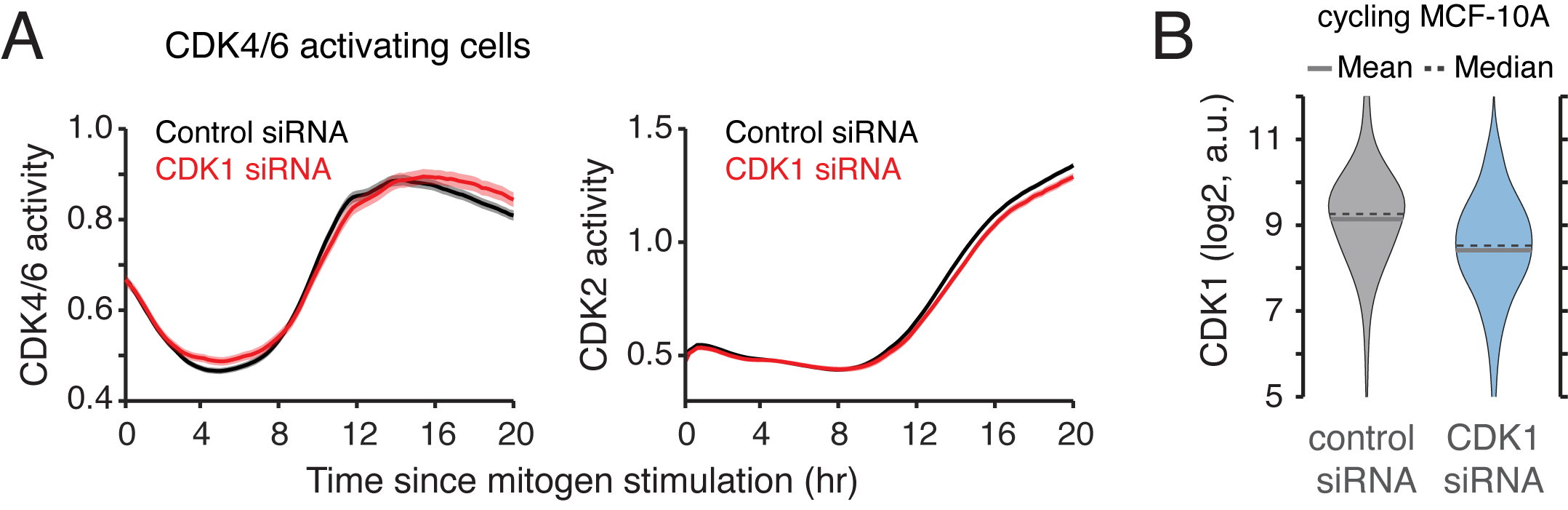
**

**Figure S1.** Effect of CDK1 knockdown on activation of the CDK2 reporter in CDK4/6 activating cells.

(A) Averaged CDK4/6 activity traces (left) and CDK2 activity traces (right) after mitogen stimulation. MCF-10A cells were transfected with control siRNA (black) and CDK1 siRNA (red) and were synchronized in quiescence by mitogen starvation for 48 hr. Data are mean ± 95% confidence interval (Control, *n* = 2,150 cells; CDK1, *n* = 1,142 cells).
(B) Expression of CDK1 protein 48 hr after siRNA transfection in cycling MCF-10A cells.

**Supplemental Figure 2**

**
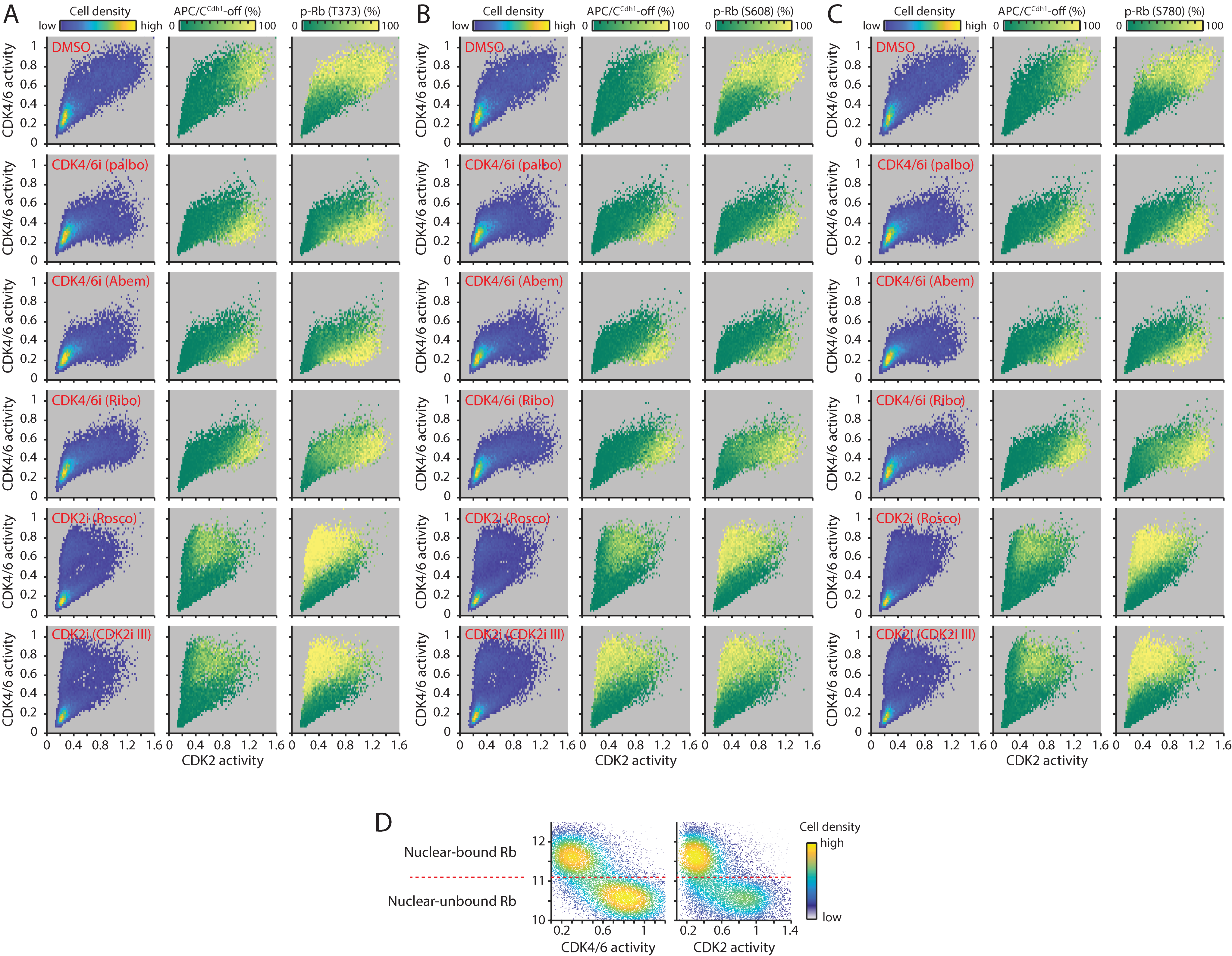
 Figure S2.** Inhibition of Rb phosphorylation by CDK4/6 inhibitors, but not by CDK2 inhibitors.

(A‒C) Three-dimensional activity maps of CDK4/6 versus CDK2 activity where cell density, the percentage of inactivated APC/C^Cdh1^, and the percentage of p-Rb at sites T373 (A), S608 (B), and S780 (C) are color-coded. After 48 hr mitogen removal, MCF-10A cells were stimulated with mitogens for 12 hr and treated with DMSO, roscovitine (60 µM), CDK2 inhibitor III (60 µM), palbociclib (1 µM), abemaciclib (1 µM) or ribociclib (1 µM) for 1 hr prior to fixation.

(D) Single-cell correlation between nuclear-bound Rb signal and CDK4/6 (left) and CDK2 (right) activity. Cell density is color-coded. After 48 hr mitogen removal, MCF-10A cells were simulated with mitogens for 12 hr prior to *in situ* extraction and fixation.

**Supplemental Figure 3**


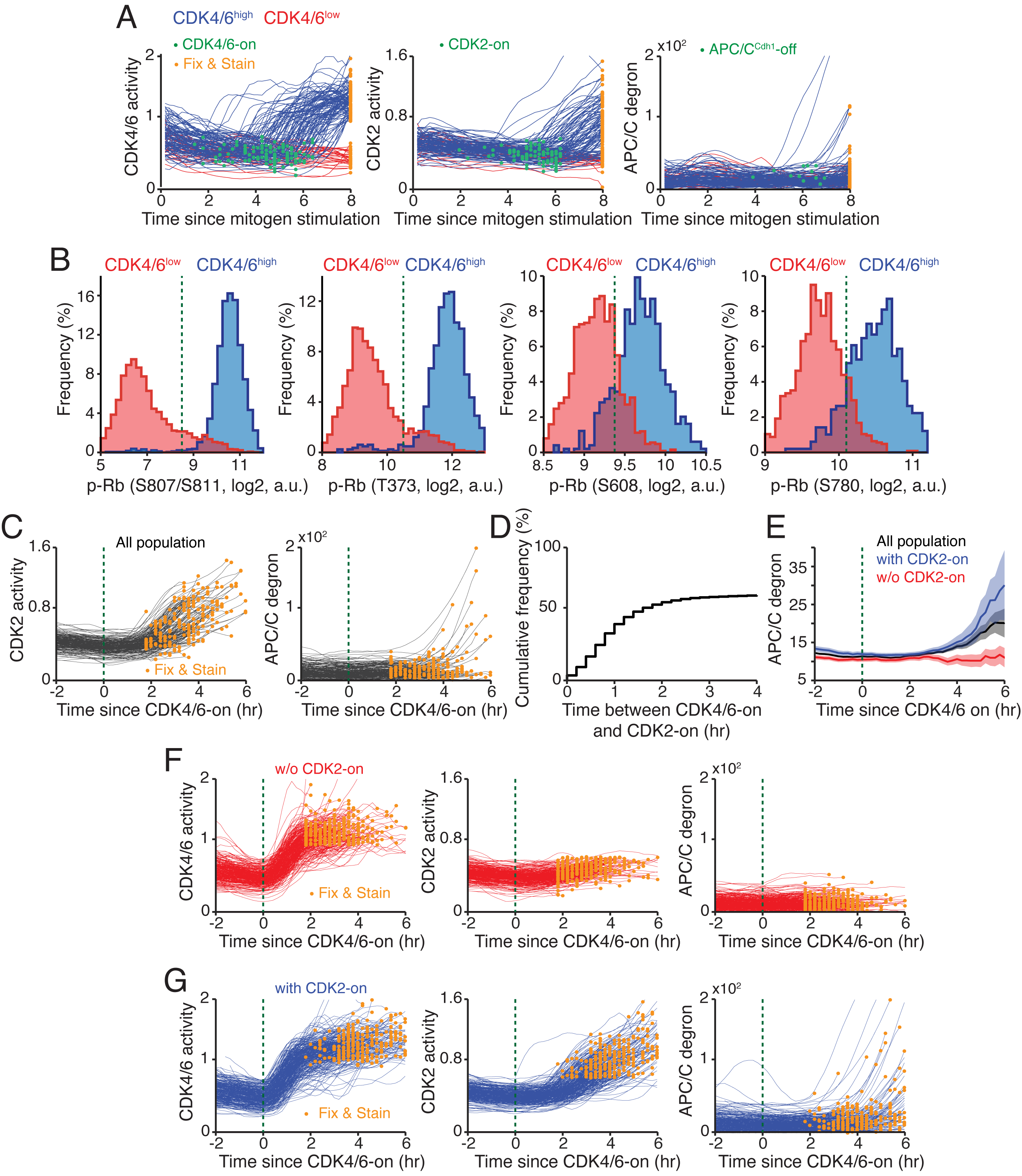


**Figure S3.** Classification of CDK4/6^high^ cells into populations with or without activated CDK2.

(A) Left to right, single-cell traces of CDK4/6 activity, CDK2 activity, and APC/C degron for CDK4/6^high^ and CDK4/6^low^ cells as a function of time after mitogen stimulation. Blue and red lines correspond to CDK2^inc^ and CDK2^low^ cells, respectively. Green and yellow points correspond to the time of activation/inactivation and fixation, respectively. To create more heterogeneity in the timing of CDK4/6 activation, mitogen-starved cells were stimulated with mitogens for various durations (9, 10, and 11 hr) prior to fixation.

(B) Left to right, distribution of p-Rb at S807/811, T373, S608, and S780 for CDK4/6^low^ (red) and CDK4/6^high^ (blue) cells. Green dotted line indicates a threshold used to classify p-Rb populations.

(C) Single-cell traces for CDK2 activity (left) and APC/C degron (right) aligned to the time of CDK4/6 activation in all population. Yellow points correspond to the time of fixation. Green dotted line indicates CDK4/6 activity onset.

(D) Cumulative frequency of time between CDK4/6 activation and CDK2 activation.

(E) Averaged APC/C degron traces aligned to the time of CDK4/6 activation. Black, blue, and red lines correspond to all population, cells with CDK2 activity, and cells without CDK2 activity, respectively. Green dotted line indicates CDK4/6 activity onset. Data are mean ± 95% confidence interval (All population, *n* = 1,917 cells; with CDK2 activity, *n* = 879 cells; w/o CDK2 activity, *n* = 1,038 cells).

(F and G) Left to right, single-cell traces of CDK4/6 activity, CDK2 activity, and APC/C degron aligned to the time of CDK4/6 activation in cells without (F) or with CDK2 activity (G). Yellow points correspond to the time of fixation. Green dotted line indicates CDK4/6 activity onset.

**Supplemental Figure 4**


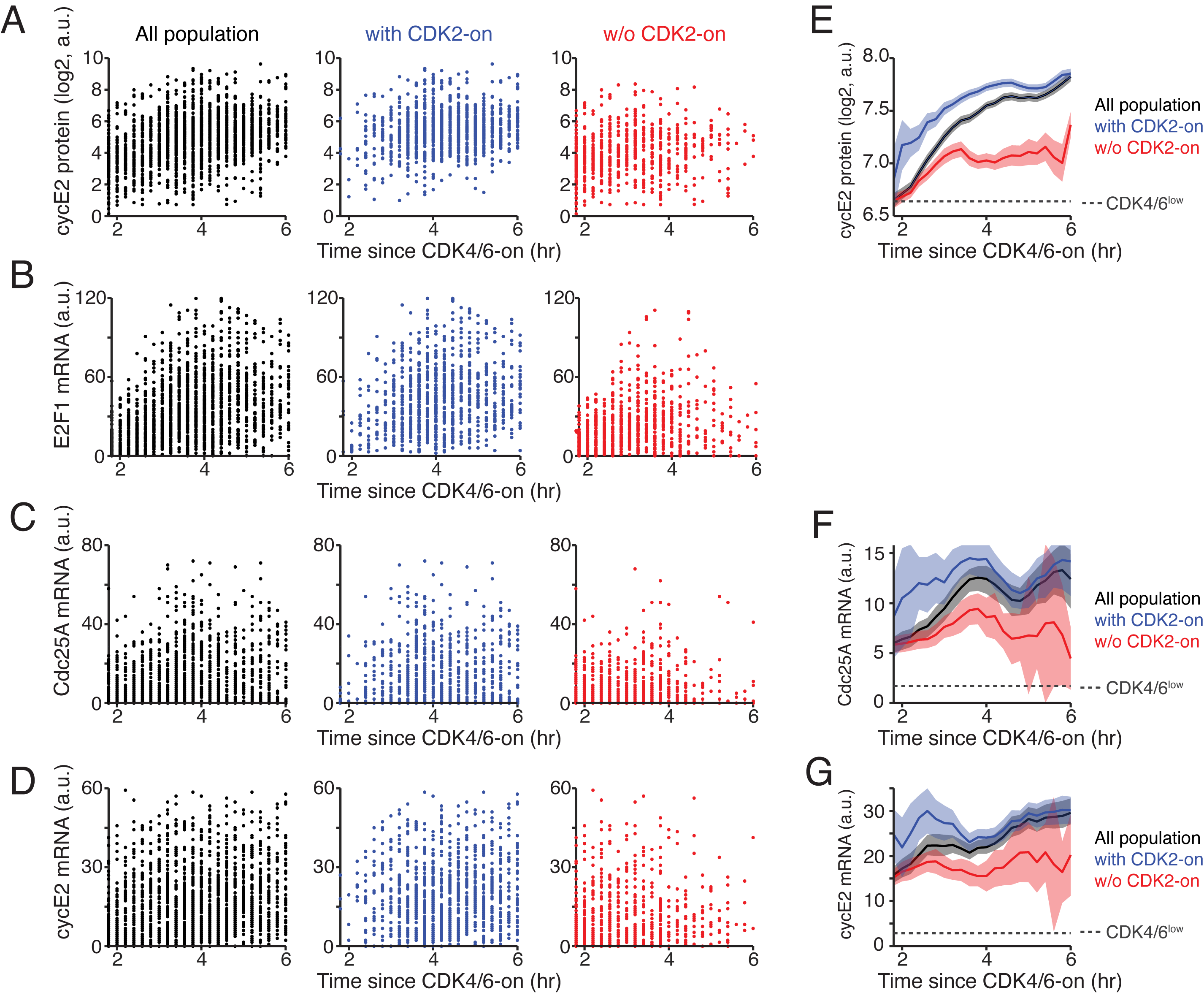


**Figure S4.** Kinetics of E2F-target gene expression as a function of time since CDK4/6 activation.

(A‒D) Cyclin E2 protein (A), E2F1 mRNA (B), Cdc25A mRNA (C), and cyclin E2 mRNA (D) levels versus time since CDK4/6 activation. Left to right, all population (black), cells with CDK2 activity (blue), and cells without CDK2 activity (red). To create more heterogeneity in the timing of CDK4/6 activation, mitogen-starved cells were stimulated with mitogens for various durations (9, 10, and 11 hr) prior to fixation.

(E‒G) Averaged cyclin E2 protein (E), Cdc25A mRNA (F), and cyclin E2 mRNA (G) levels aligned to the time of CDK4/6 activation. Black, blue, and red lines correspond to all cells, cells with CDK2 activity, and cells without CDK2 activity, respectively. Dotted line indicates protein or mRNA level in CDK4/6^low^ cells. Data are mean ± 95% confidence interval. (E: All population, *n* = 1,930 cells; with CDK2 activity, *n* = 1,226 cells; w/o CDK2 activity, *n* = 704 cells; CDK4/6^low^ cells, *n* = 3,986 cells; F: All population, *n* = 1,981 cells; with CDK2 activity, *n* = 1,068 cells; w/o CDK2 activity, *n* = 913 cells; CDK4/6^low^ cells, *n* = 4,462 cells; G: All population, *n* = 1,930 cells; with CDK2 activity, *n* = 1,226 cells; w/o CDK2 activity, *n* = 704 cells; CDK4/6^low^ cells, *n* = 3,986 cells).

**Supplemental Figure 5**


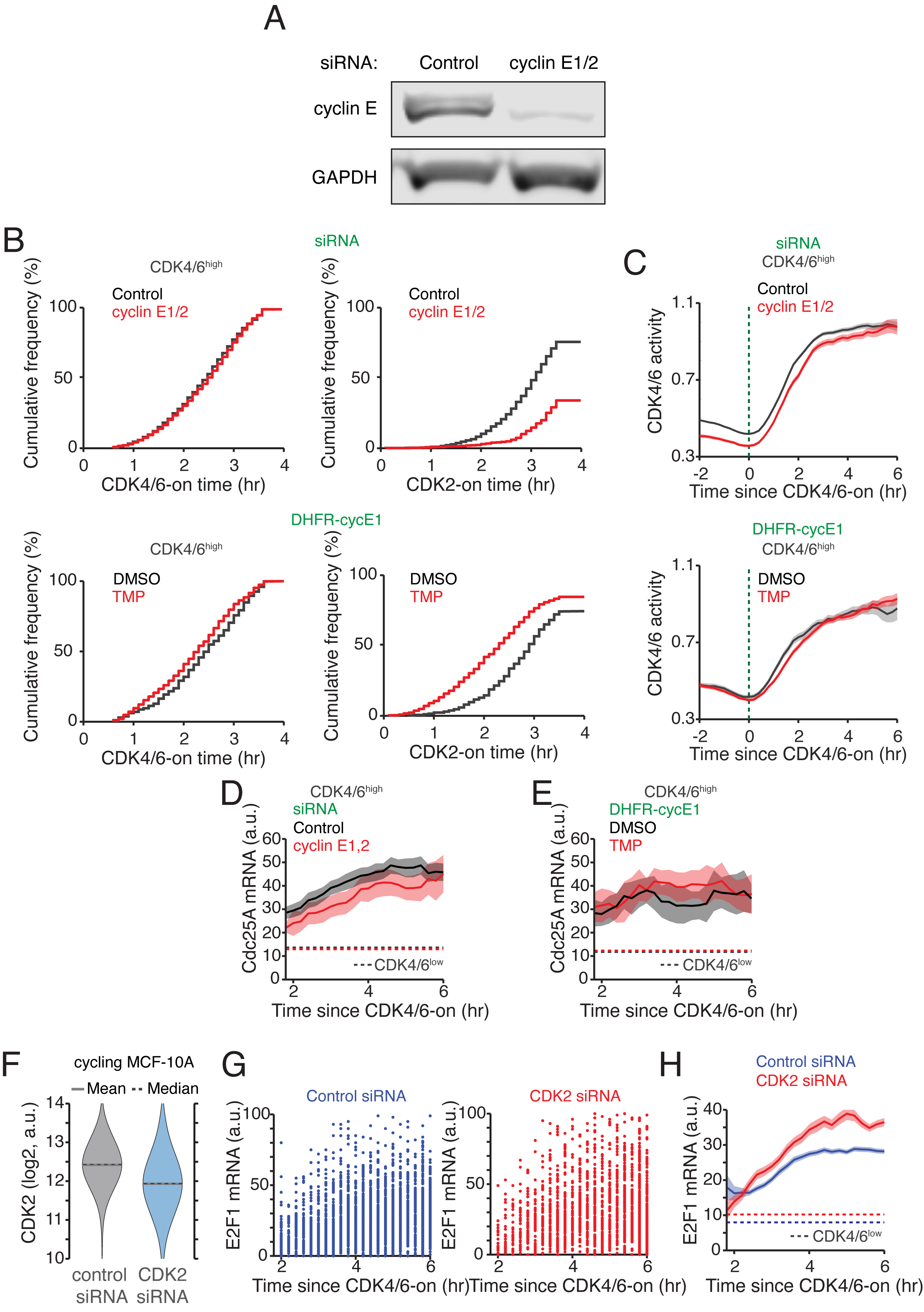


**Figure S5.** CDK4/6 activation triggers E2F activity regardless of CDK2 activation kinetics.

(A) Expression of cyclin E1/2 protein 48 hr after siRNA transfection in cycling MCF-10A cells.

(B and C) Top, MCF-10A cells were treated with control (black) and cyclin E1/2 siRNA (red). Bottom, MCF-10A cells expressing DHFR-cyclin E1 were stimulated with mitogens without (black) or with TMP (50 µM, red). Cumulative frequency of cells with CDK4/6 activation (left) and CDK2 activation (right) in CDK4/6^high^ cells (B). Average CDK4/6 activity traces aligned to the time of CDK4/6 activation in CDK4/6^high^ cells (C). Green dotted line indicates CDK4/6 activity onset. Data are mean ± 95% confidence interval (siRNA: Control, *n* = 3,172 cells; cyclin E1/2, *n* = 1,042 cells; DMSO, *n* = 470 cells; TMP, *n* = 932 cells).

(D and E) Averaged Cdc25A mRNA levels aligned to the time of CDK4/6 activation for CDK4/6^high^ cells. Dotted lines indicate averaged Cdc25A mRNA level in CDK4/6^low^ cells. MCF-10A cells were transfected with control (black) or cyclin E1/2 siRNA (red) and were stimulated with mitogens for various durations (9, 10, and 11 hr) prior to fixation (D). MCF-10A cells expressing DHFR-cyclin E1 were stimulated with mitogens for various durations (9, 10, and 11 hr) without (black) or with TMP (50 µM, red) prior to fixation (E). Data are mean ± 95% confidence interval (D, siRNA: Control, *n* = 387 cells; cyclin E1/2, *n* = 921 cells; E, DHFR-cyclin E1: DMSO, *n* = 574 cells; TMP, *n* = 495 cells).
(F) Expression of CDK2 protein 48 hr after siRNA transfection in cycling MCF-10A cells.
(G and H) E2F1 mRNA levels versus time since CDK4/6 activation (G). Averaged E2F1 mRNA levels aligned to the time of CDK4/6 activation (H). Data are mean ± 95% confidence interval. (siRNA: Control, *n* = 3,932 cells; CDK2, *n* = 2,501 cells). MCF-10A cells were transfected with control (blue) or CDK2 siRNA (red) and were stimulated with mitogens for various durations (9, 10, and 11 hr) prior to fixation.

**Supplemental Figure 6**

**
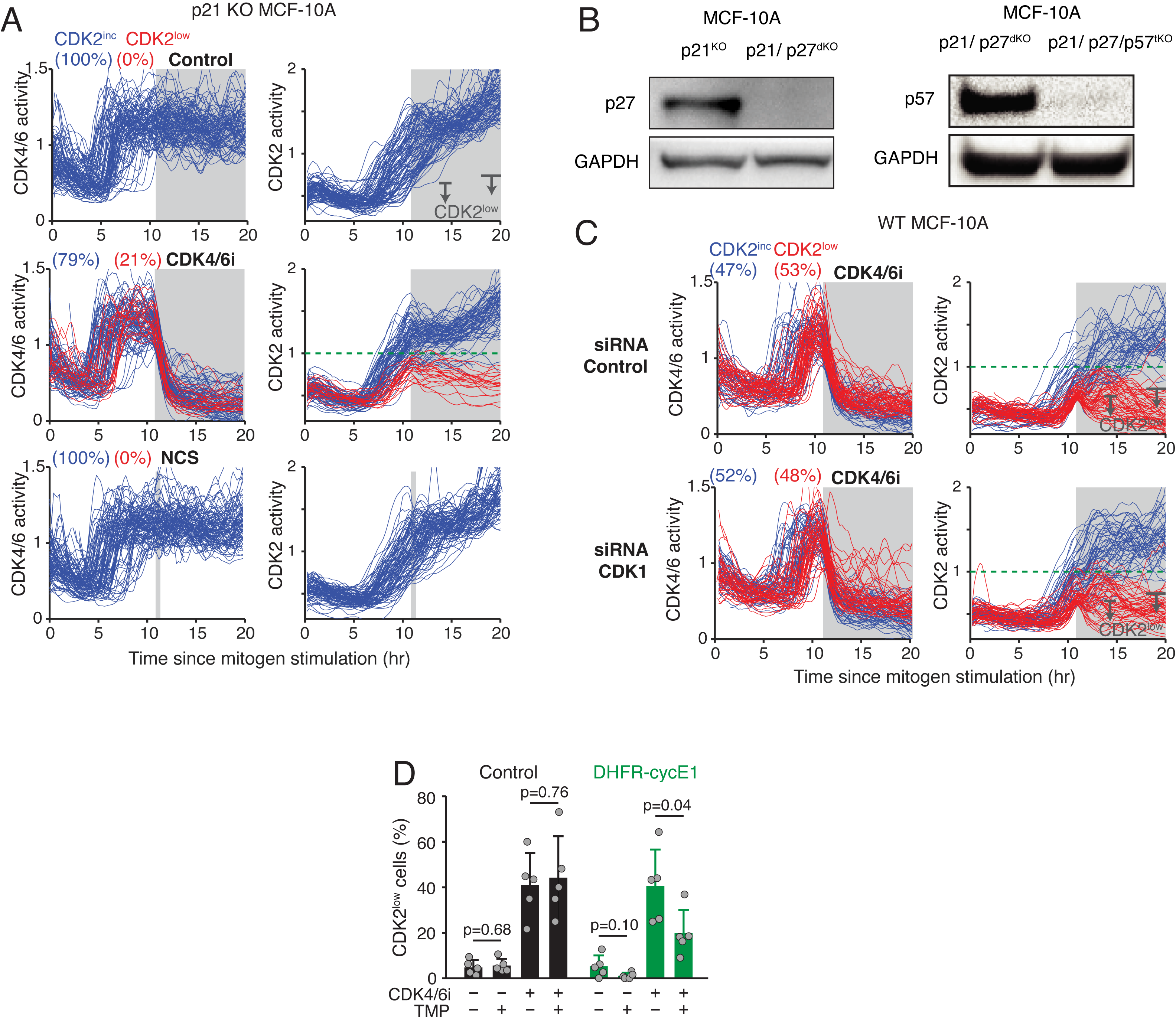
**

**Figure S6.** CDK2 activity controls CDK4/6-independent cell cycle progression.

(A) Single cell traces of CDK4/6 activity (left) and CDK2 activity (right) in p21 knockout MCF-10A cells treated with control, palbociclib (1 µM), or 10 min NCS pulse (100 ng/ml) (top to bottom). All treatments were performed 11 hr after mitogen stimulation (marked in gray). Blue and red lines correspond to CDK2^inc^ and CDK2^low^ cells, respectively.

(B) Expression of p27 in p21 KO and p21/p27 dKO MCF-10A cells (Left) and p57 in p21/p27 dKO and p21/p27/p57 tKO MCF-10A cells (right).
(C) Single cell traces of CDK4/6 activity (left) and CDK2 activity (right) in MCF-10A cells transfected with control siRNA (top) or CDK1 siRNA (bottom). MCF-10A cells were treated with palbociclib (1 µM) at 11 hr after mitogen stimulation (marked in gray). Blue and red lines correspond to CDK2^inc^ and CDK2^low^ cells, respectively. Cells were classified based on CDK2 activity using 2 windows as indicated in arrows.

(D) Percentage of CDK2^low^ cells of each condition as indicated. After 48 hr mitogen removal, MCF-10A cells without and with DHFR-cyclin E1 expression were stimulated with mitogens for 11 hr with or without TMP (50 µM) followed by acute treatment with EdU (10 µM) ± palbociclib (1 µM) for 15 min prior to fixation. Data are mean ± s.d. (*n* = 5 biological replicates). *P*-values were calculated with two-sample *t*-tests.

**Supplemental Figure 7**


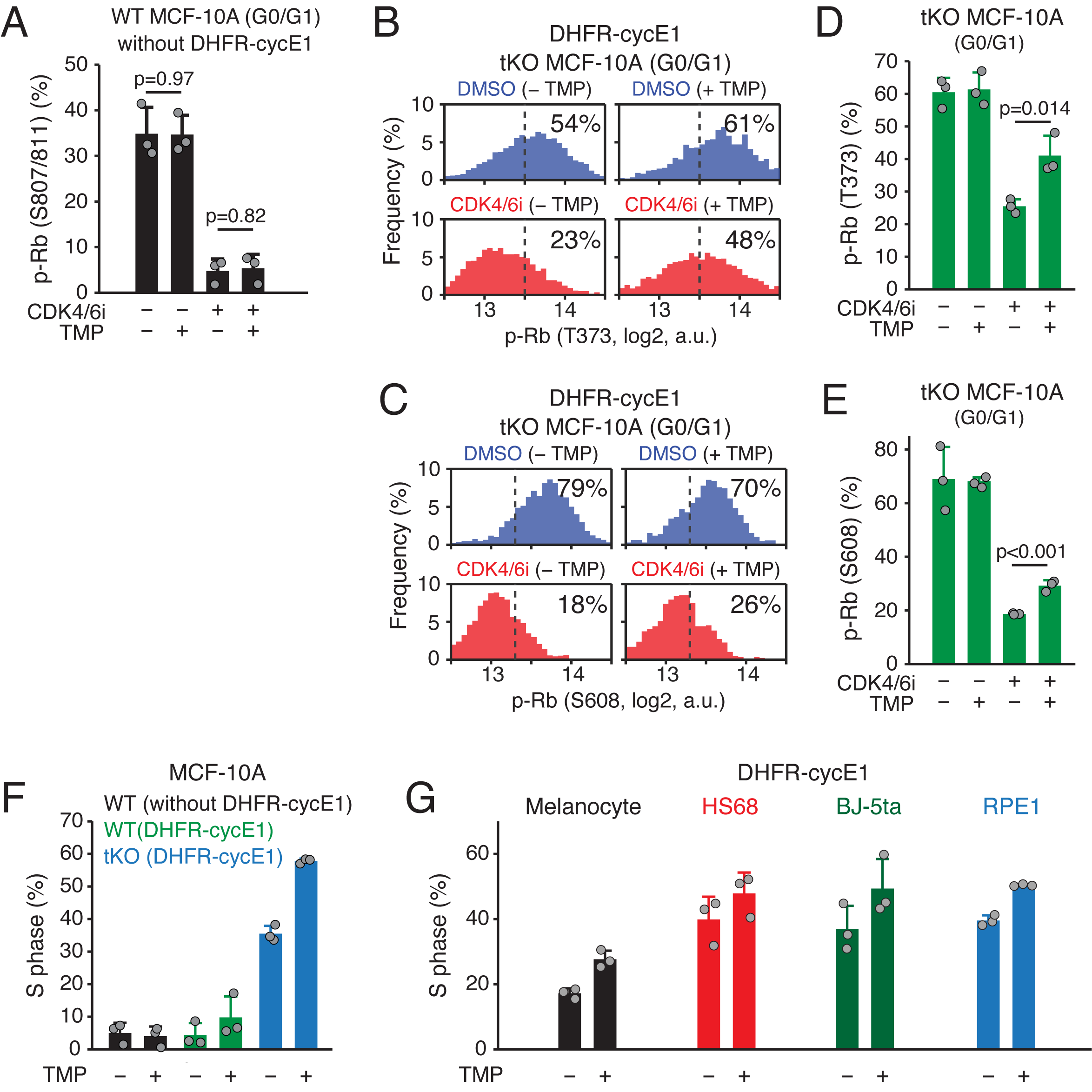


**Figure S7.** CDK2 activity regulates S phase entry.

(A) The percentage of p-Rb at S807/811 for each treatment condition as indicated. After 48 hr mitogen removal, MCF-10A cells without DHFR-cyclin E1 expression were stimulated with mitogens with or without TMP (50 µM) for 11 hr followed by acute treatment with EdU (10 µM) and palbociclib (1 µM) for 15 min prior to fixation. Data are mean ± s.d. (*n* = 3 biological replicates). *P*-values were calculated with two-sample *t*-tests.

(B–E) Histogram of p-Rb at T373 (B) and S608 (C) and the percentage of p-Rb at T373 (D) and S608 (E) for each treatment condition as indicated in p21/p27/p57 tKO MCF-10A cells with DHFR-cyclin E1 expression in G0/G1 phase. After 48 hr mitogen removal, cells were stimulated with mitogens for 11 hr with or without TMP (50 µM) followed by acute treatment with CDK4/6 inhibitor (palbociclib, 1 µM) and EdU (10 µM) for 15 min prior to fixation. Data are mean ± s.d. (*n* = 3 biological replicates). *P*-values were calculated with two-sample *t*-tests.

(F) The percentage of S phase cells for each treatment condition as indicated. After 48 hr mitogen removal, wild type MCF-10A cells without (black) and with DHFR-cyclin E1 expression (green) and p21/p27/p57 tKO MCF-10A with DHFR-cyclin E1 expression (blue) were stimulated with mitogens with or without TMP (50 μM) for 11 hr followed by acute treatment with EdU (10 μM) and palbociclib (1 μM) for 15 min prior to fixation. Data are mean ± s.d. (*n* = 3 biological replicates).

(G) Percentage of S-phase cells in Melanocyte (black), HS68 (red), BJ-5ta (green), and RPE1 (blue) cells with or without cyclin E1 overexpression. Cycling cells were treated with or without TMP for 6 hr followed by treatment with EdU (10 µM) for 15 min prior to fixation. Data are mean ± s.d. (*n* = 3 biological replicates).

**Supplemental Figure 8**


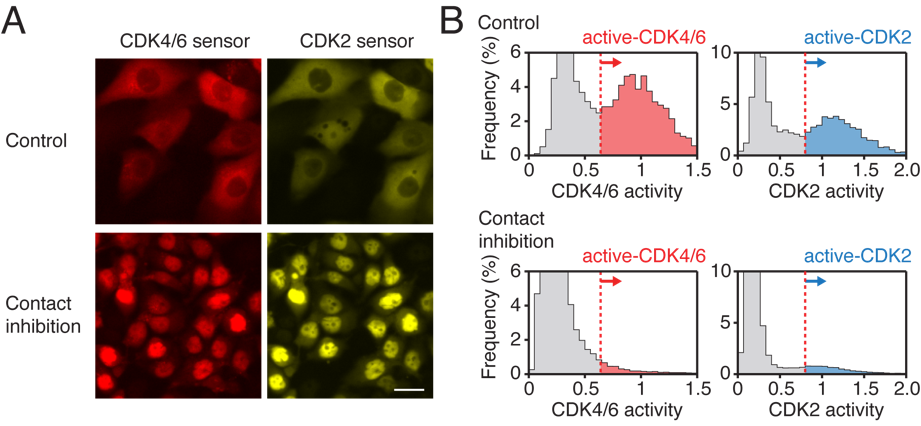


**Figure S8.** Inhibition of CDK4/6 and CDK2 activities by contact inhibition.

(A) Representative images of CDK4/6 and CDK2 reporters in MCF-10A cells in control (top) and 48-hr contact-inhibited (bottom) conditions.

(B) Histogram of CDK4/6 and CDK2 activities in control (top) and 48-hr contact-inhibited (bottom) conditions. Red dotted line indicates a threshold used to classify active CDK4/6 and CDK2 (Control, *n* = 5,266 cells; Contact inhibition, *n* = 27,499 cells).

**Supplemental Figure 9**


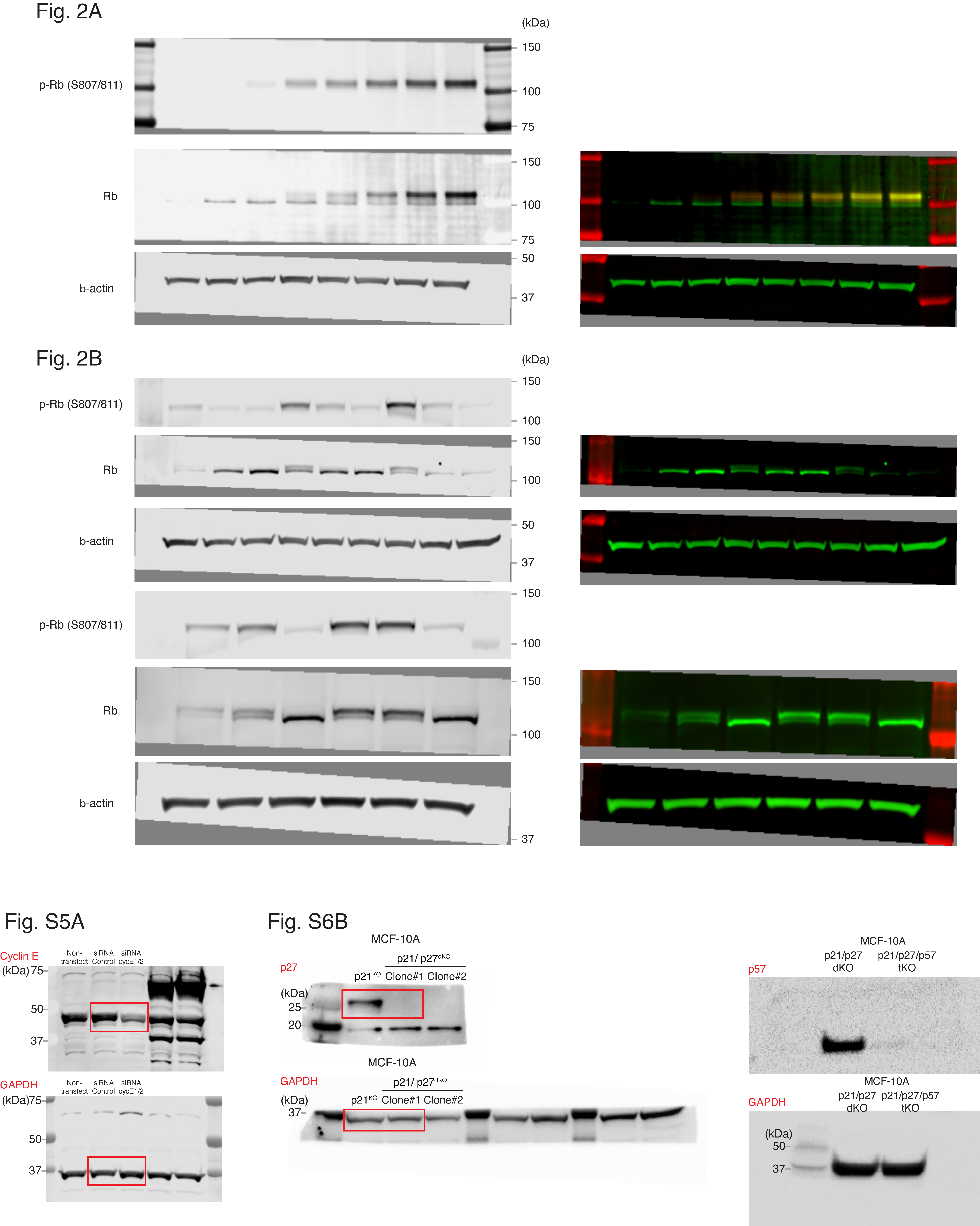


**Figure S9.** Full scan images of immunoblots.
